# The rise of state-centric biophysics: integrating orthogonal molecular readouts across biophysical scales

**DOI:** 10.1007/s12551-026-01452-9

**Published:** 2026-08-12

**Authors:** Paulo Roberto Dores-Silva, Todd Willian Pascarella, Jason Michael Newman, Júlio César Borges

**Affiliations:** 1 São Carlos Institute of Chemistry, University of São Paulo, São Carlos, SP 13566-590 Brazil; 2 Ferring Ventures Inc, 4245 Sorrento Valley Blvd, San Diego, CA 92121 USA; 3 Valenthos Biophysics, 1740 Robinson Ave, San Diego, CA 92103 USA

**Keywords:** State-centric biophysics, Multidimensional molecular characterization, Integrated biophysical platforms, Information density

## Abstract

Modern biophysics has evolved from the measurement of isolated molecular properties toward increasingly integrated molecular characterization. Advances in instrumentation, orthogonal workflows, and multidimensional analytical platforms have expanded the breadth of molecular information obtainable from a single experimental context, increasing what we define here as “information density”. In this review, we examine the scientific and technological forces underlying this transition and propose *state-centric biophysics* as an interpretive framework in which biophysical properties like affinity, kinetics, thermodynamics, stability, structural dynamics, hydrodynamics, ligand occupancy, and assembly state are viewed as complementary descriptors of a *shared molecular-state landscape* rather than as independent experimental outputs. Representative case studies, including targeted protein degradation, molecular glues, Heat Shock Protein 90 (HSP90) molecular cycle and inhibition, and state-selective inhibition of Kirsten Rat Sarcoma Viral Oncogene Homolog G12C (KRAS G12C), illustrate how biological activity frequently depends on molecular-state properties that extend beyond ligand occupancy alone. Collectively, these observations suggest that the central challenge of modern biophysics is increasingly shifting from measuring molecular properties to identifying the molecular states that govern mechanism, efficacy, selectivity, and therapeutic response.

**Supplementary Information:**

The online version contains supplementary material available at https://doi.org/10.1007/s12551-026-01452-9.

## Introduction

### The convergence of molecular measurements

Modern biophysics plays a central role in the characterization of molecular recognition, protein stability, conformational behavior, structural dynamics, and biological mechanism. Across molecular biology and drug discovery, biophysical methods are routinely used for assay development, screening, hit confirmation, mechanistic characterization, and decision-making throughout the discovery process (Holdgate et al. 2019; Renaud et al. 2016). Historically, however, most biophysical techniques were developed to interrogate specific molecular properties. Affinity, kinetics, thermodynamics, stability, structural dynamics, and molecular size were often measured independently and interpreted within the context of individual experiments (Englander and Kallenbach 1983; Konermann et al. 2011; Privalov and Potekhin 1986; Rich and Myszka 2000; Wales and Engen 2006; Wiseman et al. 1989).

The defining trend in contemporary biophysics is not the replacement of these measurements, but their increasing integration. Modern biological questions frequently require more than a single molecular parameter because molecular recognition is often coupled to changes in stability, dynamics, assembly state, hydration, conformational populations, or biological function. Consequently, confidence in molecular interpretation increasingly depends on whether complementary measurements converge toward a coherent description of the same molecular system (Holdgate et al. 2019; Renaud et al. 2016).

This transition has been accelerated by advances in instrumentation and workflow design. Automation and high-throughput experimentation expanded the scale of molecular characterization (Mayr and Fuerst 2008), while microfluidics, flow-induced dispersion analysis, integrated fluorescence workflows, electro-switchable biosensors, and mass photometry expanded the ability to characterize molecular systems through multiple complementary dimensions while reducing sample consumption (Asor and Kukura 2022; Charmet et al. 2018; Jensen and Ostergaard 2010; Langer et al. 2013; Pedersen et al. 2019; Soltermann et al. 2020). As a result, experiments increasingly generate broader molecular information from the same sample, perturbation, platform, or experimental workflow.

In this review, “*information density”* refers to the breadth and complementarity of molecular information obtainable from a single experiment, platform, sample, or tightly connected workflow. It is not intended as a formal quantitative metric, but as a conceptual descriptor of the increasing ability of modern biophysical approaches to connect biophysical properties like affinity, kinetics, thermodynamics, stability, structural dynamics, hydrodynamics, assembly state, and function within a common interpretive framework. Under this view, the value of a measurement is not determined solely by the parameter it reports, but also by how effectively it can be integrated with complementary molecular information.

The purpose of this review is to examine the convergence of molecular measurements as a defining trend in modern biophysics. We first describe the historical progression from single-parameter characterization to orthogonal workflows and integrated multidimensional platforms before discussing the scientific and technological forces that have driven this evolution. Finally, we propose *state-centric biophysics* as an interpretive framework that integrates complementary molecular measurements to better understand biological mechanism, pharmacological behavior, and therapeutic response.

An important conceptual development accompanying this experimental evolution is the recognition that biomolecules rarely exist as single static structures. Instead, proteins and molecular assemblies populate dynamic thermodynamic ensembles composed of multiple interconverting conformational states whose relative populations can be redistributed by ligand binding, post-translational modifications, environmental conditions, or molecular interactions (Boehr et al. 2009; Guo and Zhou 2016; Hammes et al. 2009; Nussinov 2025; Nussinov et al. 2023, 2026). Consequently, understanding biological function increasingly requires identifying not only whether molecular recognition occurs, but also which molecular state is being populated and how experimental perturbations alter the distribution of accessible states. This conceptual shift provides the thermodynamic foundation for the state-centric interpretive framework developed throughout this review.

### State-centric biophysics as an interpretive framework

State-centric biophysics builds upon several established areas of molecular biophysics but is not synonymous with them since they address related but distinct scientific objectives. Ensemble biophysics established that proteins and molecular assemblies exist as dynamic populations of interconverting conformations rather than as single static structures focusing on conformational populations and dynamics (Boehr et al. 2009; Nussinov 2025; Nussinov et al. 2023, 2026). Integrative structural biology combines heterogeneous experimental information to construct structural and mechanistic models of complex biological systems (Rout and Sali 2019). Orthogonal characterization workflows increase confidence by evaluating molecular systems through multiple independent experimental measurements (Holdgate et al. 2019; Renaud et al. 2016). Conceptually, state-centric biophysics focuses on identifying the experimentally supported molecular states that govern biological and pharmacological behavior.

This perspective is fundamentally grounded in thermodynamic principles, where biological function emerges from populations of interconverting molecular states rather than single static conformations. Consequently, experimental measurements become complementary observations of how molecular perturbations redistribute these state populations and influence biological behavior (Nussinov 2025; Nussinov et al. 2026).

Under this framework, the biophysical properties are interpreted as *complementary descriptors of a common molecular-state landscape*. The central question therefore shifts from whether molecules interact, whether multiple measurements agree, or whether a structural model can be constructed, to which molecular state is responsible for the observed biological outcome and how experimental perturbations redistribute populations among accessible states (Guo and Zhou 2016; Hammes et al. 2009). In this sense, state-centric biophysics is not proposed as a new experimental discipline, but as an interpretive framework for linking multidimensional molecular measurements to biological mechanism, efficacy, selectivity, and therapeutic response.

## Historical evolution of biophysical characterization

The convergence of molecular biophysical measurements emerged through a series of technological and methodological transitions that gradually expanded the scope of biophysical characterization. The history of the field is not a sequence of abrupt replacements, in which older methods were discarded as new ones appeared. Instead, successive generations of techniques added new experimental capabilities while preserving the value of established approaches. The result has been a progressive expansion from measurements focused on individual molecular properties to increasingly integrated strategies capable of describing molecular systems through multiple complementary dimensions.

### Single-parameter molecular characterization

The foundations of modern biophysical characterization were established through techniques designed to interrogate specific molecular properties with high physical rigor. During the 1980 s and early 1990 s, methods such as isothermal titration calorimetry (ITC), differential scanning calorimetry (DSC), surface plasmon resonance (SPR), hydrogen–deuterium exchange (HDX), dynamic light scattering (DLS), and analytical ultracentrifugation (AUC) became important experimental tools for studying molecular recognition, stability, dynamics, and assembly.

These techniques provided direct access to molecular properties that were often difficult to obtain by other experimental approaches. ITC enabled the determination of binding constants, stoichiometry, enthalpy, and entropy directly from solution-phase measurements (Batista et al. 2015; Jelesarov and Bosshard 1999; Wiseman et al. 1989). DSC established a rigorous framework for quantifying temperature-induced changes in proteins and macromolecular stability (Jelesarov and Bosshard 1999; Privalov and Potekhin 1986). SPR introduced real-time monitoring of biomolecular interactions, enabling kinetic and affinity measurements without the need for labeled reagents (Fagerstam et al. 1992; Jonsson et al. 1991; Pascarella et al. 2026). Hydrogen exchange methods provided a route for linking protein structure with conformational dynamics and solvent accessibility (Englander and Kallenbach 1983).

Although these approaches generated highly informative measurements, each method primarily emphasized a particular molecular dimension. Thermodynamics, kinetics, stability, size, or dynamics were typically measured independently and interpreted within the context of the individual experiment.

### Throughput expansion and automation

During the mid-1990s and early 2000 s, the focus of biophysical innovation shifted toward experimental throughput, reproducibility, and scalability. This transition was closely associated with the growing influence of pharmaceutical screening, combinatorial chemistry, and large-scale biological discovery programs.

Optical biosensors matured into robust analytical platforms, and increasing attention was directed toward improving assay quality, data analysis, and kinetic interpretation (Myszka 1999; Rich and Myszka 2000). Fluorescence-based technologies, including fluorescence polarization and fluorescence resonance energy transfer methodologies, became widely adopted because they could be implemented in automated and miniaturized assay formats (Owicki 2000). Robotic liquid handling, microplate-based experimentation, and increasingly sophisticated screening infrastructures expanded the number of experiments that could be performed within a given timeframe (Mayr and Fuerst 2008).

This period transformed the scale at which molecular characterization could be performed. Larger datasets became accessible, and the routine analysis of hundreds or thousands of samples became increasingly feasible. However, despite substantial improvements in throughput, the molecular information generated by individual experiments remained largely distributed across separate assay formats. The dominant challenge was no longer obtaining measurements, but coordinating information generated by multiple independent experimental systems.

### Orthogonal characterization workflows

As biological systems and drug-discovery programs became more complex, confidence in molecular interpretation increasingly relied on agreement across multiple independent methods. Rather than depending on a single measurement, investigators began combining complementary techniques to evaluate different aspects of the same molecular system.

This period saw the routine integration of approaches such as SPR, ITC, DSC, differential scanning fluorimetry (DSF), DLS, size-exclusion chromatography coupled to multi-angle light scattering (SEC-MALS), HDX mass spectrometry (HDX-MS), nuclear magnetic resonance (NMR) spectroscopy, X-ray crystallography, and small-angle scattering methods. NMR played a central role in revealing conformational dynamics, transient structural states, and population exchange occurring under near-physiological solution conditions, thereby establishing many of the conceptual foundations of modern ensemble-based structural biology (Guo and Zhou 2016; Weikl and Paul 2014). Complementing these capabilities, HDX-MS became particularly important by enabling direct investigation of protein dynamics, conformational changes, and solvent accessibility in solution (Englander 2006; Konermann and Scrosati 2024; Wales and Engen 2006). Together, these structural and biophysical approaches increasingly allowed molecular recognition to be interpreted within broader structural and mechanistic contexts.

The defining feature of this era was not the emergence of a new technology, but the emergence of a new experimental strategy. Confidence increasingly derived from convergence across independent measurements rather than from any individual technique alone. Reviews of biophysical methods in drug discovery emphasized the growing value of combining orthogonal approaches to support mechanistic interpretation, hit validation, and decision-making (Holdgate et al. 2019; Renaud et al. 2016).

### Integrated multidimensional platforms

Beginning in the early 2010 s, a new generation of technologies emerged that increasingly combined multiple forms of molecular characterization within the same platform or tightly connected workflow. These developments expanded the range of molecular information obtainable from limited quantities of material while reducing the dependence on large collections of independent experiments.

Flow-induced dispersion analysis demonstrated that noncovalent interactions could be quantified using nanoliter sample volumes through hydrodynamic measurements performed under native solution conditions (Jensen and Ostergaard 2010). Subsequent developments combined dispersion analysis with intrinsic fluorescence measurements to connect molecular size, folding behavior, and functional assessment within a unified workflow (Pedersen et al. 2019). Microscale thermophoresis (MST) enabled interaction analysis in free solution while remaining sensitive to changes in size, charge, hydration shell, and molecular environment (Jerabek-Willemsen et al. 2011; Seidel et al. 2013). Electro-switchable DNA-chip technologies demonstrated the simultaneous acquisition of interaction, kinetic, size, and conformational information within a single analytical framework (Langer et al. 2013).

More recently, mass photometry has enabled direct measurement of molecular masses and population distributions for individual biomolecules and complexes in solution, providing access to information related to assembly state, relative abundance, interaction equilibria, and molecular dynamics (Asor and Kukura 2022; Soltermann et al. 2020). Collectively, these developments illustrate a broader transition toward platforms that increasingly integrate complementary molecular dimensions within a common experimental context.

**Fig. 1:**
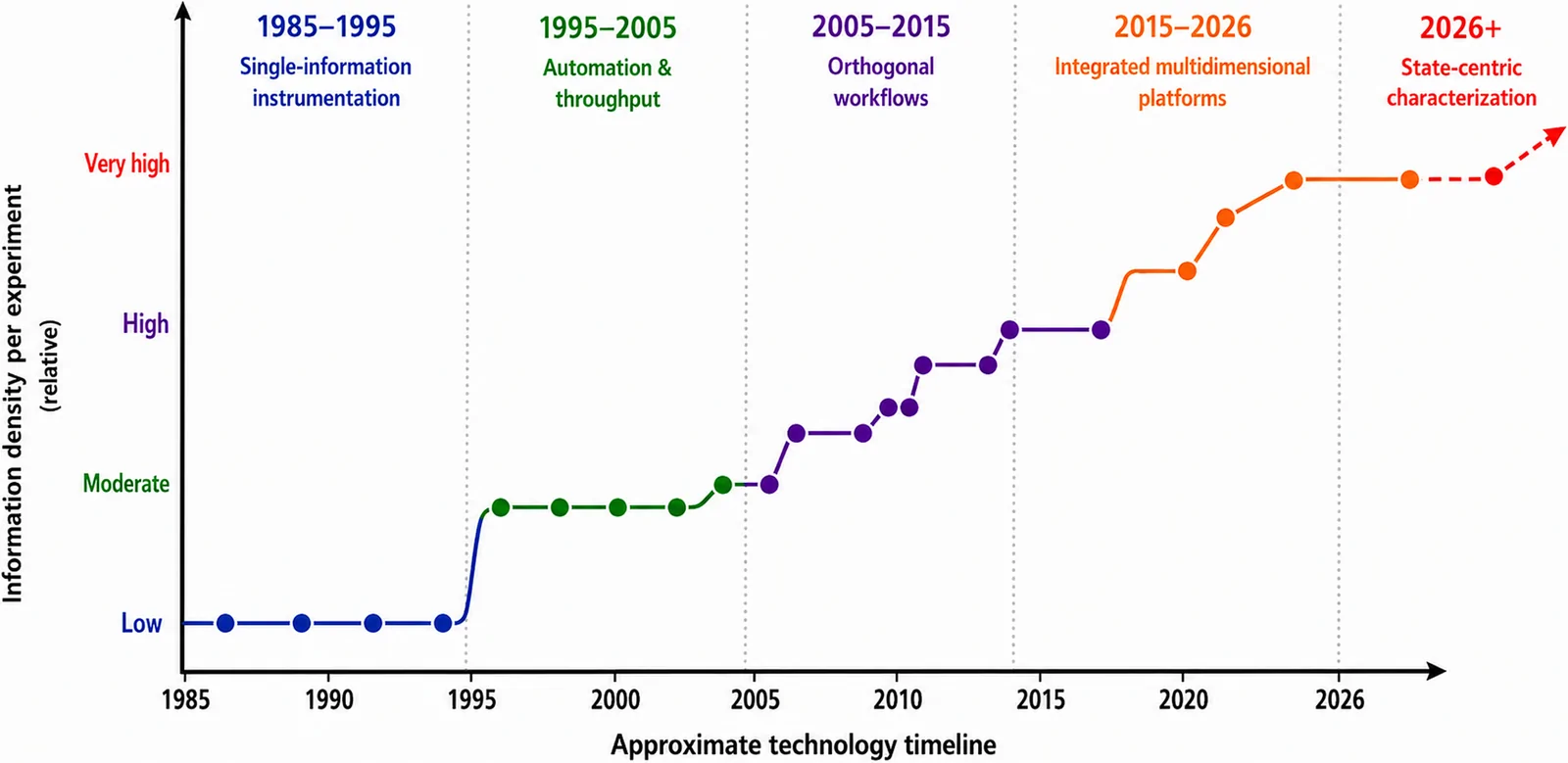
Evolution of information density in biophysical characterization and the emergence of state-centric workflows. The figure illustrates the historical progression of biophysical characterization from isolated measurements of individual molecular properties toward increasingly integrated experimental strategies capable of describing biomolecular systems through multiple complementary dimensions. The horizontal axis represents the approximate technological timeline. The vertical axis represents relative information density, defined here as the breadth and complementarity of molecular information obtainable from a single experiment, platform, sample, or tightly connected workflow. Information density is used as a conceptual descriptor rather than a formal quantitative metric. Low information density corresponds to measurements that primarily report a single molecular dimension, whereas higher information density reflects the ability to obtain multiple complementary molecular dimensions that can be interpreted together within the same experimental context or workflow. The first stage (1985–1995) was dominated by single-parameter measurements. The second stage (1995–2005) focused on automation and throughput expansion. The third stage (2005–2015) marked the widespread adoption of orthogonal characterization workflows. The fourth stage (2015–2026) reflects the emergence of integrated multidimensional platforms capable of generating multiple molecular readouts from the same sample or experimental workflow. The final stage (2026 +) represents the conceptual transition toward state-centric characterization, in which complementary molecular measurements are interpreted collectively as descriptors of a common molecular-state landscape. Collectively, the figure illustrates the progression from isolated molecular measurements toward multidimensional and state-centric interpretation

Figure 1 summarizes the historical progression of biophysical characterization from single-parameter measurements toward increasingly integrated and multidimensional experimental approaches.

### Toward integrated characterization

The historical trajectory outlined above reveals a progression from individual molecular-property measurements to orthogonal workflows and integrated platforms capable of capturing multiple molecular dimensions within increasingly unified experimental contexts. This progression established the foundation for the technological and scientific forces that continue to drive convergence across modern biophysical instrumentation. Table S1 summarizes the major stages of this evolution, highlighting the dominant paradigms, representative technologies, sources of experimental confidence, and the limitations that motivated subsequent transitions.

## Drivers of instrumentation convergence

The historical progression described in Sect. "Historical Evolution of Biophysical Characterization" did not result from a single technological breakthrough. Rather, it emerged from a combination of scientific and practical pressures that collectively reshaped modern biophysical instrumentation and experimental workflows. Increasing biological complexity, limited sample availability, the need to detect weak and heterogeneous molecular responses, growing demands for throughput, and the challenge of integrating information across multiple experimental platforms all encouraged the development of technologies capable of generating richer molecular characterization while reducing experimental burden (Holdgate et al. 2019; Mayr and Fuerst 2008; Pascarella et al. 2026; Renaud et al. 2016). As molecular systems became increasingly difficult to describe through isolated measurements, instrumentation progressively evolved toward approaches capable of capturing multiple complementary dimensions of molecular behavior within a common experimental context.

Several technological trends contributed to this transition. Miniaturization and microfluidic approaches improved sample economy and enabled quantitative characterization under sample-limited conditions (Charmet et al. 2018; Jensen and Ostergaard 2010; Otzen et al. 2021). Advances in detector sensitivity expanded access to weak, transient, and heterogeneous molecular interactions (Asor and Kukura 2022; Langer et al. 2013; Myszka and Rich 2000). Automation and parallelization increased experimental throughput and scalability for modern drug-discovery programs (Knowling et al. 2020; Mayr and Fuerst 2008; Yang et al. 2016), while solution-state characterization, orthogonal validation strategies, and developability-focused workflows broadened the range of molecular properties that could be evaluated simultaneously (Holdgate et al. 2019; Jain et al. 2017; Pedersen et al. 2019; Renaud et al. 2016; Roberts 2014). Finally, increasing computational capacity and integrative modeling approaches facilitated the interpretation of multidimensional datasets and the construction of more comprehensive molecular descriptions (Konermann et al. 2011; Rout and Sali 2019). Collectively, these developments accelerated the convergence of modern biophysical instrumentation and established the foundation for integrated and state-centric biophysical characterization strategies. The principal drivers of this transition and their impact on biophysical characterization are summarized in Table 1.

**Table 1:** From isolated measurements to multidimensional molecular characterization

Driver	Scientific/technological pressure	Instrumentation or workflow response	Impact on biophysical characterization	Representative technologies/workflows^&^	References
Miniaturization and sample economy	Many biologically relevant systems are available only in limited quantities, including recombinant targets, membrane proteins, multiprotein complexes, aggregation-prone proteins, biologics, and early drug-discovery samples	Microfluidic, capillary, nanoliter, and low-volume assay formats reduce material requirements while maintaining quantitative molecular measurements	More molecular questions can be asked from the same amount of sample; low-volume workflows support repeated, orthogonal, or multidimensional characterization	FIDA/TDA, MST, microfluidic diffusional sizing, capillary nanoDSF, microplate/capillary screening formats	(Charmet et al. 2018; Jensen and Ostergaard 2010; Otzen et al. 2021; Seidel et al. 2013)
Detector sensitivity and optical readout improvements	Modern biophysical questions often involve weak, transient, low-abundance, heterogeneous, or subtle molecular responses that require sensitive detection	Improved fluorescence, interferometric, label-free optical, and biosensor detection modalities increase sensitivity to small changes in concentration, mass, environment, diffusion, or molecular state	Lower sample concentrations and weaker interactions become experimentally accessible; subtle molecular responses can be detected with improved signal-to-noise	MST/TRIC, mass photometry, SPR/array SPR, switchSENSE, nanoDSF	(Asor and Kukura 2022; Langer et al. 2013; Rich and Myszka 2000; Seidel et al. 2013)
Automation, parallelization, and high-throughput pressure	Drug-discovery campaigns require analysis of large compound, fragment, antibody, or protein panels while maintaining reproducibility and decision speed	Robotics, multi-well assay formats, multiplexed biosensors, array-based SPR, and automated data acquisition increase experimental scale and reproducibility	Throughput increases from serial one-sample measurements toward parallelized screening and profiling; biophysical methods become more practical in discovery pipelines	HTS automation, 384/1536-well formats, Biacore 8 K, Carterra LSA, Octet/parallel BLI, plate-based fluorescence platforms	(Knowling et al. 2020; Mayr and Fuerst 2008; Renaud et al. 2016; Yang et al. 2016)
Need for solution-state and hydrodynamic context	Surface immobilization, molecular heterogeneity, oligomerization, aggregation, and conformational changes can complicate interpretation of binding-only measurements	Solution-state methods and hydrodynamic measurements increasingly complement or integrate with binding analysis	Binding can be interpreted alongside size, diffusion, hydrodynamic radius, complex formation, or aggregation behavior, improving confidence in molecular-state assignment	FIDA/TDA, SEC-MALS, DLS, mass photometry, native MS, microfluidic sizing	(Asor and Kukura 2022; Otzen et al. 2021; Pedersen et al. 2019; Pedro and Quinn 2016; Renaud et al. 2016)
Orthogonal validation and confidence in molecular interpretation	Single measurements can be insufficient to resolve whether a molecular response reflects binding, stabilization, conformational change, aggregation, avidity, or assay artifact	Biophysical workflows increasingly combine complementary methods that interrogate distinct molecular dimensions	Confidence emerges from convergence across independent readouts rather than reliance on a single parameter; experimental interpretation becomes more robust	SPR + ITC + DSF/nanoDSF + DLS/SEC-MALS + HDX-MS/native MS workflows	(Holdgate et al. 2019; Konermann et al. 2011; Renaud et al. 2016; Rich and Myszka 2000)
Biologics, developability, and stability requirements	Biologics and complex protein therapeutics require characterization of both conformational stability and colloidal behavior because aggregation, unfolding, and particle formation affect developability	Thermal profiling platforms increasingly combine intrinsic fluorescence, thermal unfolding, aggregation/backreflection, and light-scattering measurements	Thermal stability, aggregation onset, and particle-size behavior can be assessed in more integrated workflows, increasing information density for protein quality and developability studies	nanoDSF, Prometheus/Panta-style nanoDSF + backreflection + DLS/SLS, DLS/SLS platforms	(Kim et al. 2021; Renaud et al. 2016) (NanoTemperTechnologies 2025b)
Computational and data-integration capacity	Multidimensional measurements generate datasets that require integrated interpretation across structure, dynamics, thermodynamics, binding, and function	Computational modeling, structural integration, simulation, and data-fusion approaches increasingly complement experimental biophysics	Experimental readouts can be mapped into broader mechanistic models, supporting interpretation across molecular scales	HDX-MS + structural models, cryo-EM + MD, native MS + structural biology, integrative modeling workflows	(Konermann et al. 2011; Renaud et al. 2016)

^&^See Supplementary Material for List of Abbreviations

## Increasing information density per experiment

The historical evolution and the technological drivers summarized above converged toward a common outcome: modern biophysical experiments increasingly generate broader molecular information from the same sample, experimental workflow, or analytical platform. While the specific measurements obtained vary across technologies, a consistent trend has emerged across contemporary biophysics. Experiments are progressively shifting from reporting isolated molecular properties toward capturing multiple complementary dimensions of molecular behavior.

Historically, these molecular dimensions were often obtained through separate experiments and interpreted independently. The adoption of orthogonal characterization workflows expanded molecular insight by integrating complementary measurements across binding, stability, dynamics, hydrodynamics, and structure (Holdgate et al. 2019; Konermann et al. 2011; Renaud et al. 2016). More recently, integrated platforms have begun to generate multiple molecular readouts within the same experimental context, increasing the breadth of molecular information obtainable from a single sample or workflow (Asor and Kukura 2022; Jensen and Ostergaard 2010; Langer et al. 2013; Pedersen et al. 2019; Soltermann et al. 2020). Although these technologies differ substantially in physical principle, they share a common objective: expanding the molecular dimensions that can be characterized within a common experimental framework.

**Fig. 2:**
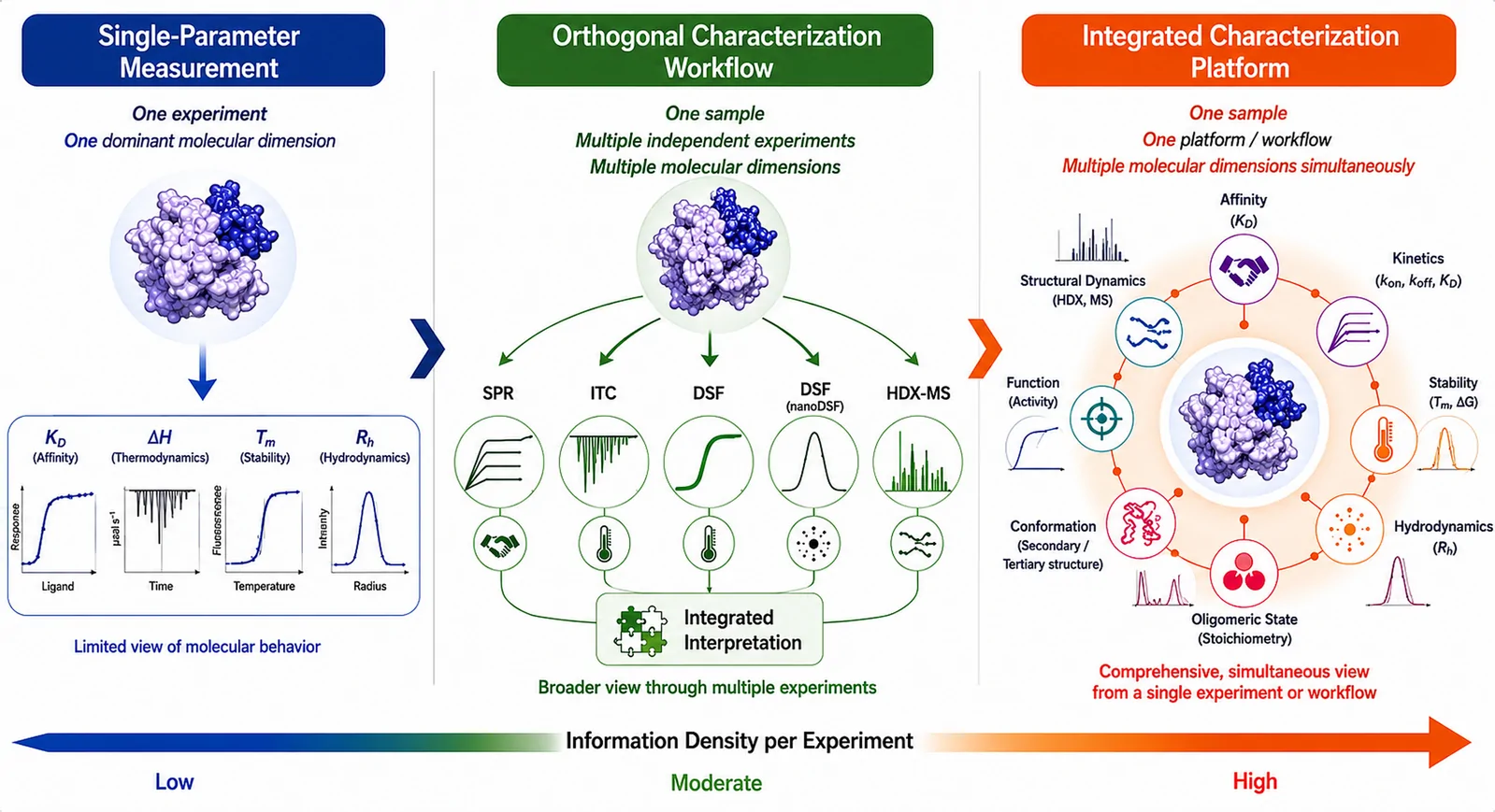
Increasing information density per experiment in modern biophysics. Traditional biophysical characterization often relied on individual experiments to interrogate a single dominant molecular dimension, such as affinity, thermodynamics, stability, or hydrodynamic behavior. Orthogonal characterization workflows subsequently combined multiple independent techniques to expand molecular insight and increase confidence in experimental interpretation. More recently, integrated platforms have begun to generate complementary molecular readouts from the same sample, perturbation, or experimental workflow, enabling simultaneous characterization of binding, kinetics, stability, hydrodynamics, conformational behavior, structural dynamics, and assembly state. This progression has increased the information density of biophysical experiments and reflects a broader transition from isolated molecular measurements toward multidimensional characterization strategies capable of describing molecular systems within a richer biological and physicochemical context

The significance of this transition extends beyond generating additional measurements. Its principal advance lies in integrating complementary molecular dimensions into coherent descriptions of molecular behavior. Rather than increasing the quantity of information alone, multidimensional characterization strengthens biological interpretation by placing affinity, stability, structural dynamics, hydrodynamics, aggregation, and assembly state within a common experimental context. This increasing information density provides part of the conceptual foundation for the state-centric framework developed in the following sections. Figure 2 summarizes this progression from single-parameter measurements to orthogonal workflows and integrated multidimensional characterization strategies, whereas Supplementary Table S2 provides a comparative overview of the corresponding experimental architectures.

## The emergence of integrated biophysical platforms

Integrated biophysical platforms vary widely in their underlying physical principles, experimental configurations, sample requirements, and molecular sensitivities. These differences make direct comparisons inappropriate and limit the extent to which results can be treated as interchangeable. Instead, each class of platform is best understood in terms of what it measures directly, what it can infer under specific conditions, and where interpretation becomes uncertain. Maintaining this distinction is critical, as integrated characterization only retains its value when the physical meaning of each measurement is preserved.

### Surface-based interaction platforms

Surface-based interaction platforms remain central to quantitative binding analysis because they report association and dissociation in real time. SPR, array SPR, biolayer interferometry, and related label-free biosensors provide direct access to kinetic parameters, including association rate constants, dissociation rate constants, residence time, and affinity when the assay is appropriately designed and modeled (Pascarella et al. 2026; Rich and Myszka 2000). Modern high-throughput biosensor formats have expanded this capability by enabling larger interaction panels, antibody characterization campaigns, and parallelized kinetic profiling (Knowling et al. 2020; Yang et al. 2016).

The principal strength of these platforms is kinetic resolution. Their primary limitation is that the molecule is usually interrogated at a surface or biosensor interface, where immobilization geometry, mass transport, rebinding, avidity, matrix effects, and model selection can influence interpretation (Pascarella et al. 2026). Complex sensorgrams can reveal heterogeneity, multiphasic behavior, or apparent conformational effects, but surface-based kinetic data alone do not provide residue-level structural information or direct oligomeric-state assignment.

Electro-switchable DNA-chip technologies, including switchSENSE and related heliX implementations, extend surface-based analysis by using electrically actuated DNA nanolevers with fluorescence detection. In addition to interaction kinetics, these platforms can report hydrodynamic diameter and conformationally sensitive changes through altered nanolever dynamics or fluorescence proximity signals (Langer et al. 2013). Applications of switchSENSE to protein-RNA and small-molecule-DNA interactions further illustrate its utility for real-time binding analysis in tethered molecular systems (Clery et al. 2017; Ramotowska et al. 2022). These measurements remain surface-coupled and tether-geometry-dependent, so hydrodynamic or conformational interpretations should be made within the constraints of the assay design.

### Thermal and stability platforms

Thermal and stability platforms interrogate molecular behavior through temperature-dependent unfolding, conformational transitions, and aggregation-related signals. DSF and nanoDSF are widely used to monitor protein stability by following fluorescence changes during thermal ramps. In nanoDSF, intrinsic tryptophan and tyrosine fluorescence can report changes in tertiary structure without the need for extrinsic dyes, making the method useful for protein stability and comparability studies, including therapeutic proteins (Wen et al. 2020).

Thermal profiling platforms are particularly valuable when conformational stability, formulation behavior, or aggregation propensity must be evaluated. NanoDSF has been applied to antibody thermal stability screening and buffer selection, supporting its use in formulation-oriented workflows (Kim et al. 2021). These approaches are also relevant to biologics because protein aggregation and instability are major developability liabilities (Roberts 2014), and clinical-stage antibody analysis has shown that developability assessment benefits from multiple biophysical parameters rather than a single stability value (Jain et al. 2017).

The interpretation caveat is important. A thermal shift can be consistent with ligand binding, altered conformational stability, or changes in aggregation behavior, but it is not automatically equivalent to affinity. Thermal readouts are most informative when the assay distinguishes conformational unfolding from colloidal instability and when thermal shifts are interpreted together with concentration dependence and appropriate controls (Llowarch et al. 2023; Niesen et al. 2007; Renaud et al. 2016). Platform configurations that combine nanoDSF with backreflection, DLS, or SLS can add aggregation or particle-size information, but these should be described according to the specific instrument configuration and assay validation (NanoTemperTechnologies 2025a).

### Solution-state and hydrodynamic platforms

Solution-state and hydrodynamic platforms provide information about molecular size, diffusion, species distribution, complex formation, and aggregation under conditions that do not require surface immobilization. Flow-induced dispersion analysis and Taylor dispersion analysis quantify molecular interactions through changes in diffusion or apparent hydrodynamic radius, enabling low-volume solution measurements of noncovalent interactions (Jensen and Ostergaard 2010). Integrated workflows combining Taylor dispersion analysis, flow-induced dispersion analysis, and intrinsic fluorescence have further connected molecular size, folding, and functional assessment within a unified protein-characterization approach (Pedersen et al. 2019). Reviews of microfluidic interaction analysis also support the value of these methods for measuring biomolecular size and binding in solution with modest sample consumption (Otzen et al. 2021).

DLS provides hydrodynamic information by measuring diffusion-dependent scattering fluctuations. It is useful for assessing sample homogeneity, aggregation, and apparent particle size in solution, but it is highly sensitive to large particles and polydispersity (Stetefeld et al. 2016). SEC-MALS adds a complementary dimension by separating species chromatographically before multi-angle light scattering analysis, enabling molecular-weight and size characterization of monomers, oligomers, aggregates, complexes, glycoproteins, and detergent-associated membrane-protein systems when separation and sample quality are adequate (Batista et al. 2015; Some et al. 2019).

The key strength of hydrodynamic platforms is that they help define the physical species present during an experiment. Their limitation is that size, diffusion, or scattering changes do not by themselves identify a binding site or molecular mechanism. These methods are strongest when used to establish whether the measured species is monomeric, oligomeric, aggregated, complexed, or heterogeneous under the assay conditions.

### Conformation-sensitive platforms

Conformation-sensitive binding platforms detect interaction-associated changes in fluorescence, thermal gradients, or local dye environment. MST measures changes in thermophoretic movement that can arise from binding-associated differences in size, charge, hydration shell, or conformation (Jerabek-Willemsen et al. 2011; Seidel et al. 2013). Because the assay is performed in free solution and can tolerate complex buffers, MST can be useful for systems that are difficult to immobilize or analyze by surface-based methods.

Temperature-related intensity change (TRIC), Dianthus, and Spectral Shift implementations extend fluorescence-based affinity analysis into plate-compatible or high-throughput formats. The safest interpretation is that these methods quantify concentration-dependent binding-associated fluorescence responses. Recent review literature describes Spectral Shift as a tool for studying macromolecular interactions and discusses Dianthus as a plate-based, immobilization-free, high-throughput platform for protein–ligand, protein–protein, protein-nucleic acid, and ternary-complex assays, with TRIC as an orthogonal fluorescence-based implementation (Hunter et al. 2025).

These approaches are powerful but should not be overinterpreted. A fluorescence shift or thermophoretic response indicates a binding-associated change in the measured signal; it does not automatically specify the structural origin of that change. Dye labeling, local environmental effects, compound fluorescence, aggregation, adsorption, and buffer mismatch can all influence the response. Therefore, conformation-sensitive platforms are best described as affinity and binding-response methods with conditional sensitivity to molecular environment, not as standalone structural methods.

### Structural and ensemble platforms

Structural and ensemble platforms add molecular dimensions that are not captured by affinity or stability measurements alone. HDX-MS measures backbone amide exchange as a function of solvent accessibility and hydrogen-bonding dynamics, providing information on structural dynamics, conformational protection, ligand-induced changes, and allosteric effects (Konermann et al. 2011). Because HDX-MS reports ensemble-averaged exchange behavior and depends on peptide coverage, back-exchange control, spatial resolution, and data analysis choices, interpretation requires rigorous experimental and reporting standards (Masson et al. 2019).

Native mass spectrometry preserves noncovalent complexes sufficiently to analyze ligand binding, stoichiometry, complex composition, and assembly state for suitable systems. It has been used in fragment-based drug discovery to detect weak ligand binding and characterize stoichiometry and affinity under native-like conditions (Pedro and Quinn 2016). Ion mobility mass spectrometry adds gas-phase separation according to ion mobility and collision cross section, providing information related to the size and shape of protein ions and complexes (Christofi and Barran 2023). These approaches are powerful for composition and assembly analysis, but gas-phase transfer, ionization, adduct formation, and preservation of native-like states must be carefully controlled and interpreted.

Mass photometry provides a label-free optical approach for measuring molecular mass distributions and relative abundances of individual biomolecules and complexes in solution. Molecular counting by mass photometry can quantify protein–protein interactions and species distributions under appropriate concentration and mass-resolution conditions (Soltermann et al. 2020). Applications to membrane proteins further illustrate its value for heterogeneous and assembly-state-sensitive systems (Olerinyova et al. 2021), and recent reviews position mass photometry as a tool for studying biomolecular interactions, affinities, and dynamics (Asor and Kukura 2022).

Finally, integrative structural biology provides the conceptual and computational framework for combining heterogeneous experimental data into coherent structural models. Integrative studies combine information from multiple experimental methods and prior knowledge while explicitly accounting for uncertainty and resolution (Rout and Sali 2019). In the context of biophysical characterization, such approaches are most useful when structural, dynamic, mass, and interaction data must be interpreted together rather than treated as separate experimental outputs.

Collectively, these technologies differ in what they measure directly, what they infer conditionally, and what they cannot resolve without additional evidence. Table 2 summarizes the molecular dimensions captured by representative integrated biophysical platforms and distinguishes direct readouts from conditional or interpretation-dependent readouts.

**Table 2:** Major readout classes and interpretation limits of integrated biophysical platforms

Platform class	Direct readouts	Conditional readouts	Major interpretation caution	Representative references
SPR/array SPR/BLI	Affinity; association and dissociation kinetics; residence time	Heterogeneity; avidity; conformational effects	Surface immobilization, mass transport, rebinding, matrix effects, and model selection can bias interpretation	(Knowling et al. 2020; Pascarella et al. 2026; Rich and Myszka 2000; Yang et al. 2016)
FIDA/TDA workflows	Hydrodynamic radius; diffusivity; affinity from binding-dependent size changes	Folding changes; oligomerization; complex formation	Requires detectable size or diffusivity changes and appropriate assay design	(Jensen and Ostergaard 2010; Otzen et al. 2021; Pedersen et al. 2019)
switchSENSE/heliX	Affinity; kinetics; hydrodynamic diameter	Conformational change; ternary or multivalent complexes	DNA-nanolever attachment, surface geometry, and tethering must be considered	(Clery et al. 2017; Langer et al. 2013; Ramotowska et al. 2022)
nanoDSF/Prometheus-type thermal platforms	Thermal stability; aggregation or particle-size readouts when configured	Ligand binding inferred from stabilization or destabilization	Thermal shifts are not automatically equivalent to affinity; aggregation and irreversibility can complicate interpretation	(Jain et al. 2017; Kim et al. 2021; Roberts 2014; Wen et al. 2020)
Dianthus/TRIC/Spectral Shift	Affinity from concentration-dependent fluorescence responses	Binding-associated environmental or conformational sensitivity	Dye environment, compound fluorescence, aggregation, adsorption, and buffer mismatch require controls	(Hunter et al. 2025; Jerabek-Willemsen et al. 2011; Seidel et al. 2013)
Mass photometry	Molecular mass distributions; relative abundance; assembly state	Affinity estimates; population dynamics	Requires suitable concentration range, mass resolution, and control of surface interactions	(Asor and Kukura 2022; Olerinyova et al. 2021; Soltermann et al. 2020)
Native MS/ion mobility MS	Stoichiometry; ligand occupancy; complex composition; assembly state	Conformational heterogeneity; affinity ranking	Gas-phase transfer, ionization, adducts, and preservation of native-like states must be controlled	(Christofi and Barran 2023; Gavriilidou et al. 2022; Pedro and Quinn 2016)
HDX-MS	Structural dynamics; solvent accessibility; conformational protection; allosteric perturbation	Binding interfaces; stabilization patterns; assembly interfaces	Peptide coverage, back-exchange, spatial resolution, ensemble averaging, and data analysis limit atomistic interpretation	(Englander and Kallenbach 1983; Konermann et al. 2011; Masson et al. 2019; Wales and Engen 2006)

A more detailed comparison of direct, derived, and conditional readouts is provided in Supplementary Table S3.

## Toward state-centric biophysics

Contemporary biophysics is shifting from the measurement of isolated molecular properties toward integrated, multidimensional descriptions of molecular systems. Advances in instrumentation, combined with increasingly connected characterization platforms and orthogonal workflows, now make it possible to evaluate affinity, kinetics, thermodynamics, stability, structural dynamics, hydrodynamics, and assembly state within unified experimental contexts (Holdgate et al. 2019; Renaud et al. 2016). In this framework, molecular recognition is no longer adequately described by a single parameter; instead, binding must be understood in relation to the broader physical state of the system under investigation.

### Beyond affinity

Affinity remains one of the most important descriptors of molecular recognition. Equilibrium dissociation constants quantify the free-energy difference between bound and unbound states and remain fundamental to both mechanistic studies and drug discovery. However, affinity alone rarely provides a complete description of molecular behavior. Two interactions with identical equilibrium affinities may exhibit markedly different association rates, dissociation rates, residence times, thermodynamic signatures, conformational consequences, or biological outcomes (Copeland et al. 2006; Decherchi and Cavalli 2020; Schuetz et al. 2017).

Affinity also does not directly report whether binding is coupled to conformational change, altered stability, oligomerization, aggregation, redistribution of conformational populations, or changes in assembly state. Consequently, a binding constant represents an important description of molecular recognition, but it does not necessarily identify the molecular state in which that recognition occurs or define the physical condition of the molecular system being interrogated.

The concept of a molecular state extends beyond a single structural representation of a biomolecule. Proteins, nucleic acids, molecular complexes, and higher-order assemblies rarely exist as static entities. Instead, they populate ensembles of interconverting states that differ in structure, dynamics, stability, interaction competence, assembly state, and functional behavior (Boehr et al. 2009; Nussinov et al. 2023). A molecular state can therefore be defined as a physically distinguishable condition of a molecular system characterized by a particular combination of structural organization, conformational population, dynamic behavior, thermodynamic stability, assembly state, and interaction competence (Guo and Zhou 2016; Nussinov et al. 2023).

Consequently, molecular recognition is often more accurately described as a transition between molecular states rather than as a simple interaction between static molecular structures. Binding events may stabilize pre-existing conformations, redistribute conformational populations, promote assembly formation, alter dynamic behavior, or shift equilibria among multiple accessible states (Guo and Zhou 2016; Hammes et al. 2009).

**Fig. 3:**
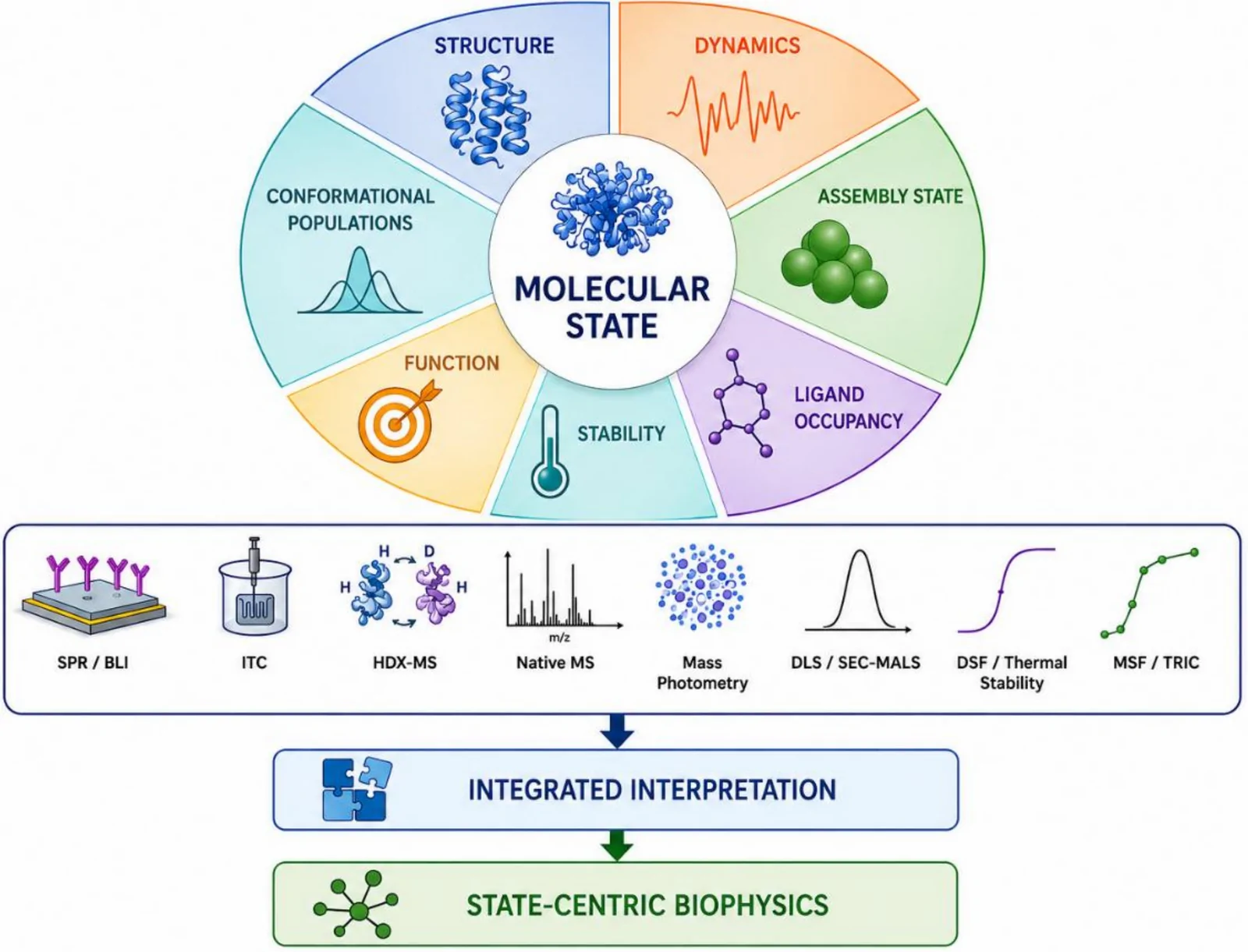
Molecular states as multidimensional descriptions of biomolecular systems and their integration through state-centric biophysics. Molecular recognition occurs within molecular systems that are defined by multiple interconnected physical and functional dimensions rather than by a single experimental parameter. In this framework, a molecular state is represented by the combined contributions of structure, dynamics, assembly state, ligand occupancy, thermodynamic stability, biological function, and conformational population distributions. These dimensions collectively define the physical condition of a molecular system and determine how that system responds to biological or pharmacological perturbation. Different biophysical methods provide complementary observations of these dimensions. Individually, each measurement captures only part of the molecular-state landscape. Collectively, they provide the experimental context required to define the molecular state being interrogated. State-centric biophysics emerges from the integration of these complementary observations into a coherent molecular description. Rather than interpreting affinity, stability, dynamics, assembly state, or structure independently, this framework seeks to identify the molecular states that govern biological function and to understand how experimental perturbations redistribute populations among accessible states

Figure 3 illustrates this concept by depicting molecular states as multidimensional entities defined by interconnected structural, dynamic, thermodynamic, assembly-state, and functional properties. Within this framework, affinity, kinetics, thermodynamics, stability, hydrodynamics, conformational populations, and assembly state are viewed as complementary descriptors of the same underlying molecular reality rather than independent experimental observations.

### Molecular recognition as population redistribution

The modern view of biomolecular behavior recognizes that proteins and molecular assemblies exist as dynamic ensembles rather than as single static structures (Boehr et al. 2009; Nussinov 2025; Nussinov et al. 2023, 2026; Weikl and Paul 2014). Under these conditions, molecular recognition is frequently associated with changes in state populations. Binding may stabilize a pre-existing conformation, shift equilibrium distributions, alter structural dynamics, promote assembly formation, or modulate allosteric communication pathways (Guo and Zhou 2016; Hammes et al. 2009). Consequently, the biological significance of an interaction often depends not only on whether binding occurs, but also on how that interaction redistributes populations among accessible molecular states.

This perspective helps explain why interactions with similar apparent affinities can produce markedly different biological outcomes. Molecular recognition may occur within folded monomers, transient intermediates, oligomeric assemblies, heterogeneous populations, or dynamically fluctuating conformational ensembles, each associated with distinct functional consequences. Affinity, kinetics, stability, dynamics, and assembly state therefore become interconnected manifestations of the same molecular system rather than independent descriptors of molecular behavior.

### State-centric interpretation

State-centric biophysics provides an interpretive framework for integrating complementary molecular measurements into experimentally supported descriptions of molecular behavior. Rather than evaluating individual biophysical parameters in isolation, it seeks to determine which molecular state governs biological function and how experimental perturbations redistribute populations among accessible states.

Modern biophysical methods contribute complementary information toward this objective. Structural-dynamics approaches such as HDX-MS reveal conformational protection, flexibility, and allosteric perturbation (Konermann et al. 2011; Masson et al. 2019). Hydrodynamic measurements identify changes in molecular size and complex formation (Jensen and Ostergaard 2010; Otzen et al. 2021; Pedersen et al. 2019). Native MS, SEC-MALS, and mass photometry characterize stoichiometry, assembly state, and population distributions (Asor and Kukura 2022; Pedro and Quinn 2016; Soltermann et al. 2020; Some et al. 2019), whereas stability measurements reveal changes in conformational robustness and aggregation propensity (Janin 2010; Roberts 2014). Collectively, these complementary observations provide the experimental context required to define the molecular state being interrogated.

State-centric biophysics does not replace traditional biophysical measurements. Rather, it provides a framework for interpreting them collectively. As multidimensional characterization becomes increasingly accessible, the focus of interpretation shifts from determining whether binding occurs to determining which molecular state is being measured and how experimental perturbations redistribute populations among accessible states.

## Impact on drug discovery and biological research

The emergence of multidimensional and state-centric biophysics has implications that extend beyond instrumentation, experimental design, and data integration. The value of these approaches ultimately lies in their ability to improve the interpretation of complex molecular systems. This impact becomes particularly apparent when biological targets exhibit weak binding, conformational heterogeneity, dynamic assembly behavior, or mechanistic complexity that cannot be adequately described through isolated measurements. In these contexts, the ability to place molecular recognition within a broader state-dependent framework can substantially influence experimental interpretation and decision-making.

### Complex targets, molecular assemblies, and state-dependent recognition

Many of the most challenging systems in modern biology and drug discovery do not behave as simple rigid molecular entities. Intrinsically disordered proteins, multidomain signaling proteins, membrane proteins, induced-proximity systems, molecular glues, transient complexes, and higher-order molecular assemblies exist as dynamic populations rather than as single well-defined structures (Boehr et al. 2009; Luo et al. 2023; Nussinov et al. 2023; Renaud et al. 2016). Under these conditions, molecular recognition is often coupled to changes in conformation, oligomerization, assembly state, dynamics, or population distributions. Consequently, the biological significance of an interaction may depend as much on the molecular state of the system as on the interaction itself.

This complexity becomes particularly apparent in weak and transient interactions, where distinguishing productive molecular recognition from nonspecific association, aggregation, avidity effects, or assay-dependent artifacts can be challenging (Coyle and Walser 2020; Robson-Tull 2018). Similarly, conformational heterogeneity can produce situations in which ligands with similar apparent affinities stabilize different conformational populations and therefore generate different biological outcomes (Guo and Zhou 2016; Nussinov et al. 2023). Molecular assemblies introduce an additional layer of complexity because stoichiometry, assembly state, subunit exchange, and population distributions can influence biological behavior independently of affinity measurements alone (Asor and Kukura 2022; Gavriilidou et al. 2022; Pedro and Quinn 2016; Soltermann et al. 2020).

From a practical perspective, the value of multidimensional biophysics is not that it eliminates uncertainty, but that it helps identify the molecular origin of uncertainty. Complementary measurements can reveal whether a molecular response is associated with conformational stabilization, altered dynamics, assembly-state redistribution, oligomerization, aggregation, or other state-dependent phenomena (Holdgate et al. 2019; Renaud et al. 2016). Confidence therefore emerges not from the number of measurements collected, but from the coherence of the molecular description generated by those measurements. This principle forms the foundation for the real-world examples discussed below.

### Real-world examples of state-centric interpretation

The practical value of state-centric biophysics becomes most apparent when integrated measurements reveal mechanistic features that cannot be inferred from affinity alone. Similar interaction measurements may therefore correspond to fundamentally different molecular states and biological outcomes when examined through complementary experimental dimensions. The following examples illustrate how multidimensional characterization provides mechanistic insights that extend beyond conventional affinity-based interpretation.

One illustrative example is provided by proteolysis-targeting chimeras (PROTACs). Classical binary affinity measurements quantify interactions between a PROTAC and its individual protein partners. However, subsequent structural, kinetic, and degradation studies demonstrated that biological activity depends strongly on the properties of the ternary complex formed between the target protein, the PROTAC, and the recruited E3 ligase (Gadd et al. 2017; Roy et al. 2019). In the case of the Bromodomain and Extra-Terminal (BET) degrader MZ1, structural studies revealed highly cooperative ternary-complex formation despite modest binary binding affinities. SPR measurements further demonstrated that ternary-complex dissociation kinetics correlated more strongly with degradation efficiency than binary affinity alone. Consequently, similar affinity measurements could lead to markedly different biological outcomes because the relevant molecular state was defined by ternary-complex stability, cooperativity, and residence time rather than by binary binding strength.

A second example emerges from molecular-glue degraders. Traditional affinity-based interpretation would predict activity primarily from direct binding between individual molecular partners. However, studies combining native MS and mass photometry demonstrated that molecular glues can remodel oligomeric equilibria and alter assembly-state populations within E3 ligase systems (Huang et al. 2023). In these cases, the biological mechanism was not adequately described by a single binding interaction. Instead, the compounds promoted redistribution among alternative assembly states, producing functional outcomes that depended on changes in complex composition and population abundance. The relevant molecular state therefore involved assembly architecture and oligomeric organization rather than affinity alone.

A third example is provided by Heat Shock Protein 90 (HSP90) inhibitor characterization. Many HSP90 ligands exhibit comparable binding affinities despite producing distinct biological effects. Integrative studies combining kinetic measurements, structural analysis, molecular simulations, and machine-learning approaches demonstrated that ligands with similar equilibrium affinities can stabilize different conformational ensembles and engage distinct allosteric communication pathways (Blacklock and Verkhivker 2014; Minari et al. 2019; Morra et al. 2009; Verkhivker et al. 2020). Under a purely affinity-centered interpretation, these ligands would appear mechanistically similar. However, multidimensional characterization revealed that differences in conformational dynamics, kinetic behavior, and allosteric regulation contributed substantially to their biological activity. The relevant molecular state therefore extended beyond simple ligand occupancy to include conformational and dynamic features of the HSP90 ensemble.

A fourth example is provided by covalent Kirsten Rat Sarcoma Viral Oncogene Homolog Gly12Cys mutant (KRAS G12C) inhibitors. Early medicinal chemistry efforts frequently focused on affinity optimization as the primary route toward improved target engagement. However, structural, biochemical, and cellular studies revealed that successful KRAS G12C inhibitors such as sotorasib and adagrasib derive their activity from selective engagement of a specific nucleotide-dependent conformational state of KRAS (Canon et al. 2019; Hallin et al. 2020; Ostrem et al. 2013). These inhibitors bind preferentially to the inactive GDP-bound form of KRAS G12C and exploit a transient switch-II pocket that is not equivalently accessible across all conformational states. Consequently, biological activity depends not only on ligand affinity but also on conformational-state populations, nucleotide cycling, and the dynamic redistribution of KRAS between accessible structural states. From a state-centric perspective, these compounds do not simply bind KRAS; they selectively engage and stabilize a therapeutically relevant molecular state within the broader conformational landscape of the protein.

Collectively, these examples demonstrate that the biological significance of molecular recognition frequently depends on properties that extend beyond ligand occupancy alone. Ternary-complex stability, assembly-state redistribution, conformational populations, kinetic persistence, and allosteric communication can all contribute to biological outcome, even when interactions exhibit similar apparent affinities. Consequently, affinity remains an essential descriptor of molecular recognition, but it does not necessarily identify the molecular state responsible for biological activity (Guo and Zhou 2016; Hammes et al. 2009).

Viewed together, these case studies illustrate how multidimensional characterization can connect molecular recognition to the molecular states that govern mechanism, efficacy, selectivity, and therapeutic response. From this perspective, the value of integrated biophysics lies not simply in generating additional measurements and information density, but in providing the experimental context required to identify and interpret biologically relevant molecular states.

## Future directions

The progression described throughout this review reflects a broader transformation in the role of biophysics within biomolecular research and drug discovery. Historically, advances in biophysical instrumentation focused on improving sensitivity, throughput, precision, or experimental accessibility. More recently, however, the emphasis has shifted toward the integration of complementary molecular information within increasingly connected experimental frameworks. The future of the field will likely be defined not only by the development of new technologies, but by the ability to combine diverse measurements into coherent and reproducible descriptions of molecular systems.

### Next-generation integrated platforms

Future generations of biophysical instrumentation will likely continue the trend toward increasingly integrated experimental workflows. The most significant advances may not arise from entirely new physical principles, but from tighter coupling between complementary measurements that are currently performed separately.

Recent developments already illustrate this direction. Structural prediction methods based on artificial intelligence have dramatically expanded the ability to generate structural hypotheses for proteins, protein complexes, nucleic acids, and small-molecule interactions (Abramson et al. 2024; Jumper et al. 2021). At the same time, experimental platforms continue to evolve toward multidimensional characterization, where binding, stability, dynamics, hydrodynamics, and assembly state can be evaluated within increasingly unified workflows.

Importantly, computational prediction and experimental biophysics should not be viewed as competing approaches. Predictive models generate hypotheses, whereas experimental measurements establish whether those hypotheses accurately describe the molecular system under investigation. Future integrated platforms will likely derive much of their value from the ability to connect these two capabilities more effectively.

### Increasing information density

The concept of information density introduced earlier in this review provides a useful framework for understanding the ongoing evolution of biophysical characterization. Future technological development will likely continue to increase the diversity of molecular dimensions that can be interrogated within the same experimental context. However, the value of these advances will depend not only on the quantity of information generated, but also on the ability to preserve the physical meaning of individual measurements while integrating them into coherent molecular descriptions. Consequently, future multidimensional platforms will be judged not simply by the amount of data they generate, but by their ability to support mechanistic interpretation across multiple dimensions of molecular behavior.

### Remaining technical challenges

Despite substantial progress, important challenges remain. Many biophysical measurements remain sensitive to sample quality, heterogeneity, aggregation, concentration effects, surface interactions, model assumptions, and experimental design. Different platforms often interrogate different aspects of the same system and may not always produce immediately consistent interpretations.

Multidimensional characterization introduces additional complexity because integrating multiple measurements can also integrate multiple sources of uncertainty. Experimental disagreement may arise from differences in timescale, molecular population sensitivity, detection principle, or physical context rather than from experimental error alone. As a result, future progress will require improved strategies for identifying, quantifying, communicating and treating uncertainty across experimental workflows.

Another challenge involves the interpretation of increasingly complex datasets. Methods such as HDX-MS, native MS, mass photometry, and integrative structural approaches can generate information-rich datasets that require sophisticated computational analysis and careful experimental controls (Konermann and Scrosati 2024; Masson et al. 2019). Continued advances in data analysis, validation strategies, and uncertainty assessment will therefore remain essential.

### Standardization, interoperability, and data fusion

The long-term success of multidimensional biophysics may depend as much on standardization as on instrumentation. As datasets become increasingly heterogeneous, reproducible interpretation requires common standards for reporting, metadata capture, uncertainty estimation, and data exchange.

Efforts in HDX-MS have already demonstrated the value of community-driven recommendations for experimental design, interpretation, and reporting (Masson et al. 2019). Similarly, integrative structural biology has developed frameworks for combining heterogeneous experimental information while maintaining transparency regarding assumptions, uncertainties, and model quality (Rout and Sali 2019). Initiatives such as PDB-Dev and the IHM-CIF standard further illustrate how data standards and specialized repositories can support reproducible integrative modeling workflows (Vallat et al. 2021, 2024).

The FAIR principles provide an additional foundation by emphasizing that scientific data should be findable, accessible, interoperable, and reusable (Wilkinson et al. 2016). These concepts are likely to become increasingly important as multidimensional characterization generates larger and more interconnected datasets.

The continued growth of multidimensional biophysics will require experimental, computational, and community frameworks that support reproducible integration of heterogeneous molecular data. Standardization, interoperability, transparent uncertainty assessment, and accessible data infrastructures will therefore become increasingly important as biophysical characterization expands across experimental scales and disciplines.

## Conclusions

The progression described throughout this review reflects a broader transformation in how molecular systems are experimentally characterized and interpreted. Advances in instrumentation, orthogonal workflows, and integrated characterization platforms have expanded the breadth of molecular information that can be obtained from a single experimental context, enabling molecular recognition to be examined through multiple complementary biophysical dimensions rather than isolated measurements.

These developments support a shift from interpreting the biophysical properties as independent experimental outputs toward viewing them as complementary descriptors of a common molecular-state landscape. In this review, we propose state-centric biophysics as an interpretive framework for integrating these measurements into experimentally supported descriptions of the molecular states that govern biological mechanism, efficacy, selectivity, and therapeutic response.

Ultimately, the future impact of multidimensional biophysics will depend not simply on generating additional data, but on improving our ability to identify, characterize, and interpret the molecular states that determine biological function. As experimental characterization continues to evolve, the central challenge of modern biophysics is increasingly shifting from measuring molecular properties to understanding the molecular states that underlie biological behavior.

## Figure preparation

All conceptual figures were conceived, designed, scientifically curated, and validated by the authors. Artificial intelligence-assisted image generation (OpenAI image generation models, 2026 release) was used solely as a graphical production tool to generate visual elements following detailed author-defined prompts and iterative refinement. The final figure composition, scientific content, organization, labeling, and interpretation were determined entirely by the authors. Final publication-quality figures were generated by exporting the artwork as high-resolution raster images using Inkscape v1.4.4 and subsequently enhancing image resolution using Topaz Gigapixel AI v1.3.3 (Topaz Labs, Dallas, TX, USA; Art & CG and Recover v3 models) to improve visual clarity for publication. Artificial intelligence was used exclusively as a graphical production and image-enhancement tool and did not contribute to the scientific concepts, interpretation, analysis, or conclusions presented in the figures.

## Supplementary Information

Below is the link to the electronic supplementary material.

ESM 1 (PDF 95.1 KB )

## Data Availability

No datasets were generated or analysed during the current study.

## References

[CR1] Abramson J, Adler J, Dunger J, Evans R, Green T, Pritzel A, Ronneberger O, Willmore L, Ballard AJ, Bambrick J et al (2024) Accurate structure prediction of biomolecular interactions with AlphaFold 3. Nature 630:493–500. 10.1038/s41586-024-07487-w PMC11168924 38718835

[CR2] Asor R, Kukura P (2022) Characterising biomolecular interactions and dynamics with mass photometry. Curr Opin Chem Biol 68:102132. 10.1016/j.cbpa.2022.102132 35405425

[CR3] Batista FA, Gava LM, Pinheiro GM, Ramos CH, Borges JC (2015) From conformation to interaction: techniques to explore the Hsp70/Hsp90 network. Curr Protein Pept Sci 16:735–753. 10.2174/1389203716666150505225744 25961397

[CR4] Blacklock K, Verkhivker GM (2014) Computational modeling of allosteric regulation in the hsp90 chaperones: a statistical ensemble analysis of protein structure networks and allosteric communications. PLoS Comput Biol 10:e1003679. 10.1371/journal.pcbi.1003679 PMC4055421 24922508

[CR5] Boehr DD, Nussinov R, Wright PE (2009) The role of dynamic conformational ensembles in biomolecular recognition. Nat Chem Biol 5:789–796. 10.1038/nchembio.232 PMC2916928 19841628

[CR6] Canon J, Rex K, Saiki AY, Mohr C, Cooke K, Bagal D, Gaida K, Holt T, Knutson CG, Koppada N et al (2019) The clinical KRAS(G12C) inhibitor AMG 510 drives anti-tumour immunity. Nature 575:217–223. 10.1038/s41586-019-1694-1 31666701

[CR7] Charmet J, Arosio P, Knowles TPJ (2018) Microfluidics for protein biophysics. J Mol Biol 430:565–580. 10.1016/j.jmb.2017.12.015 29289566

[CR8] Christofi E, Barran P (2023) Ion mobility mass spectrometry (IM-MS) for structural biology: insights gained by measuring mass, charge, and collision cross section. Chem Rev 123:2902–2949. 10.1021/acs.chemrev.2c00600 PMC10037255 36827511

[CR9] Clery A, Sohier TJM, Welte T, Langer A, Allain FHT (2017) switchSENSE: a new technology to study protein-RNA interactions. Methods 118–119:137–145. 10.1016/j.ymeth.2017.03.004 28286323

[CR10] Copeland RA, Pompliano DL, Meek TD (2006) Drug-target residence time and its implications for lead optimization. Nat Rev Drug Discov 5:730–739. 10.1038/nrd2082 16888652

[CR11] Coyle J, Walser R (2020) Applied biophysical methods in fragment-based drug discovery. SLAS Discov 25:471–490. 10.1177/2472555220916168 32345095

[CR12] Decherchi S, Cavalli A (2020) Thermodynamics and kinetics of drug-target binding by molecular simulation. Chem Rev 120:12788–12833. 10.1021/acs.chemrev.0c00534 PMC8011912 33006893

[CR13] Englander SW (2006) Hydrogen exchange and mass spectrometry: a historical perspective. J Am Soc Mass Spectrom 17:1481–1489. 10.1016/j.jasms.2006.06.006 PMC3427778 16876429

[CR14] Englander SW, Kallenbach NR (1983) Hydrogen exchange and structural dynamics of proteins and nucleic acids. Q Rev Biophys 16:521–655. 10.1017/s0033583500005217 6204354

[CR15] Fagerstam LG, Frostell-Karlsson A, Karlsson R, Persson B, Ronnberg I (1992) Biospecific interaction analysis using surface plasmon resonance detection applied to kinetic, binding site and concentration analysis. J Chromatogr 597:397–410. 10.1016/0021-9673(92)80137-j 1517343

[CR16] Gadd MS, Testa A, Lucas X, Chan KH, Chen W, Lamont DJ, Zengerle M, Ciulli A (2017) Structural basis of PROTAC cooperative recognition for selective protein degradation. Nat Chem Biol 13:514–521. 10.1038/nchembio.2329 PMC5392356 28288108

[CR17] Gavriilidou AFM, Sokratous K, Yen HY, De Colibus L (2022) High-throughput native mass spectrometry screening in drug discovery. Front Mol Biosci 9:837901. 10.3389/fmolb.2022.837901 PMC9047894 35495635

[CR18] Guo J, Zhou HX (2016) Protein allostery and conformational dynamics. Chem Rev 116:6503–6515. 10.1021/acs.chemrev.5b00590 PMC5011433 26876046

[CR19] Hallin J, Engstrom LD, Hargis L, Calinisan A, Aranda R, Briere DM, Sudhakar N, Bowcut V, Baer BR, Ballard JA et al (2020) The KRAS(G12C) inhibitor MRTX849 provides insight toward therapeutic susceptibility of KRAS-mutant cancers in mouse models and patients. Cancer Discov 10:54–71. 10.1158/2159-8290.CD-19-1167 PMC6954325 31658955

[CR20] Hammes GG, Chang YC, Oas TG (2009) Conformational selection or induced fit: a flux description of reaction mechanism. Proc Natl Acad Sci U S A 106:13737–13741. 10.1073/pnas.0907195106 PMC2728963 19666553

[CR21] Holdgate G, Embrey K, Milbradt A, Davies G (2019) Biophysical methods in early drug discovery. ADMET DMPK 7:222–241. 10.5599/admet.733 PMC8963580 35359617

[CR22] Huang X, Kamadurai H, Siuti P, Ahmed E, Bennett JL, Donald WA (2023) Oligomeric remodeling by molecular glues revealed using native mass spectrometry and mass photometry. J Am Chem Soc 145:14716–14726. 10.1021/jacs.3c02655 37379266

[CR23] Hunter CE, Pohl E, Freitag-Pohl S (2025) High-throughput investigation of macromolecular interactions for drug development using spectral shift technology. Biophys Rev 17:959–980. 10.1007/s12551-025-01359-x PMC12686307 41378104

[CR24] Jain T, Sun T, Durand S, Hall A, Houston NR, Nett JH, Sharkey B, Bobrowicz B, Caffry I, Yu Y et al (2017) Biophysical properties of the clinical-stage antibody landscape. Proc Natl Acad Sci U S A 114:944–949. 10.1073/pnas.1616408114 PMC5293111 28096333

[CR25] Janin YL (2010) ATPase inhibitors of heat-shock protein 90, second season. Drug Discov Today 15:342–353. 10.1016/j.drudis.2010.03.002 20230904

[CR26] Jelesarov I, Bosshard HR (1999) Isothermal titration calorimetry and differential scanning calorimetry as complementary tools to investigate the energetics of biomolecular recognition. J Mol Recognit 12:3–18. 10.1002/(sici)1099-1352(199901/02)12:1<3::Aid-jmr441>3.0.Co;2-6 10398392

[CR27] Jensen H, Ostergaard J (2010) Flow induced dispersion analysis quantifies noncovalent interactions in nanoliter samples. J Am Chem Soc 132:4070–4071. 10.1021/ja100484d 20201527

[CR28] Jerabek-Willemsen M, Wienken CJ, Braun D, Baaske P, Duhr S (2011) Molecular interaction studies using microscale thermophoresis. Assay Drug Dev Technol 9:342–353. 10.1089/adt.2011.0380 PMC3148787 21812660

[CR29] Jonsson U, Fagerstam L, Ivarsson B, Johnsson B, Karlsson R, Lundh K, Lofas S, Persson B, Roos H, Ronnberg I et al (1991) Real-time biospecific interaction analysis using surface plasmon resonance and a sensor chip technology. Biotechniques 11:620 627. https://pubmed.ncbi.nlm.nih.gov/1804254/1804254

[CR30] Jumper J, Evans R, Pritzel A, Green T, Figurnov M, Ronneberger O, Tunyasuvunakool K, Bates R, Zidek A, Potapenko A et al (2021) Highly accurate protein structure prediction with AlphaFold. Nature 596:583–589. 10.1038/s41586-021-03819-2 PMC8371605 34265844

[CR31] Kim SH, Yoo HJ, Park EJ, Na DH (2021) Nano differential scanning fluorimetry-based thermal stability screening and optimal buffer selection for immunoglobulin G. Pharmaceuticals (Basel) 15:29. 10.3390/ph15010029 PMC8778976 35056086

[CR32] Knowling S, Clark J, Sjuts H, Abdiche YN (2020) Direct comparison of label-free biosensor binding kinetics obtained on the Biacore 8K and the Carterra LSA. SLAS Discov 25:977–984. 10.1177/2472555220934814 32646263

[CR33] Konermann L, Scrosati PM (2024) Hydrogen/deuterium exchange mass spectrometry: fundamentals, limitations, and opportunities. Mol Cell Proteomics 23:100853. 10.1016/j.mcpro.2024.100853 PMC11570944 39383946

[CR34] Konermann L, Pan J, Liu YH (2011) Hydrogen exchange mass spectrometry for studying protein structure and dynamics. Chem Soc Rev 40:1224–1234. 10.1039/c0cs00113a 21173980

[CR35] Langer A, Hampel PA, Kaiser W, Knezevic J, Welte T, Villa V, Maruyama M, Svejda M, Jahner S, Fischer F et al (2013) Protein analysis by time-resolved measurements with an electro-switchable DNA chip. Nat Commun 4:2099. 10.1038/ncomms3099 PMC3719012 23839273

[CR36] Llowarch P, Usselmann L, Ivanov D, Holdgate GA (2023) Thermal unfolding methods in drug discovery. Biophys Rev (Melville) 4:021305. 10.1063/5.0144141 PMC10903397 38510342

[CR37] Luo S, Wohl S, Zheng W, Yang S (2023) Biophysical and integrative characterization of protein intrinsic disorder as a prime target for drug discovery. Biomolecules 13. 10.3390/biom13030530 PMC10046839 36979465

[CR38] Masson GR, Burke JE, Ahn NG, Anand GS, Borchers C, Brier S, Bou-Assaf GM, Engen JR, Englander SW, Faber J et al (2019) Recommendations for performing, interpreting and reporting hydrogen deuterium exchange mass spectrometry (HDX-MS) experiments. Nat Methods 16:595–602. 10.1038/s41592-019-0459-y PMC6614034 31249422

[CR39] Mayr LM, Fuerst P (2008) The future of high-throughput screening. J Biomol Screen 13:443–448. 10.1177/1087057108319644 18660458

[CR40] Minari K, de Azevedo EC, Kiraly VTR, Batista FAH, de Moraes FR, de Melo FA, Nascimento AS, Gava LM, Ramos CHI, Borges JC (2019) Thermodynamic analysis of interactions of the Hsp90 with adenosine nucleotides: a comparative perspective. Int J Biol Macromol. 10.1016/j.ijbiomac.2019.02.116 30797004

[CR41] Morra G, Verkhivker G, Colombo G (2009) Modeling signal propagation mechanisms and ligand-based conformational dynamics of the Hsp90 molecular chaperone full-length dimer. PLoS Comput Biol 5:e1000323. 10.1371/journal.pcbi.1000323 PMC2649446 19300478

[CR42] Myszka DG (1999) Improving biosensor analysis. J Mol Recognit 12:279–284. 10.1002/(SICI)1099-1352(199909/10)12:5<279::AID-JMR473>3.0.CO;2-3 10556875

[CR43] Myszka DG, Rich RL (2000) Implementing surface plasmon resonance biosensors in drug discovery. Pharm Sci Technol Today 3:310–317. 10.1016/s1461-5347(00)00288-1 10996572

[CR44] NanoTemperTechnologies (2025a) nano Differential Scanning Fluorimetry (nanoDSF). https://nanotempertech.com/nanodsf/. Accessed 06252026

[CR45] NanoTemperTechnologies (2025b) Prometheus™ Panta: one readout. Multiple insights. https://nanotempertech.com/prometheus/?utm. Accessed 25 June 2026

[CR46] Niesen FH, Berglund H, Vedadi M (2007) The use of differential scanning fluorimetry to detect ligand interactions that promote protein stability. Nat Protoc 2:2212–2221. 10.1038/nprot.2007.321 17853878

[CR47] Nussinov R (2025) Pioneer in molecular biology: conformational ensembles in molecular recognition, allostery, and cell function. J Mol Biol 437:169044. 10.1016/j.jmb.2025.169044 PMC12021580 40015370

[CR48] Nussinov R, Liu Y, Zhang W, Jang H (2023) Protein conformational ensembles in function: roles and mechanisms. RSC Chem Biol 4:850–864. 10.1039/d3cb00114h PMC10619138 37920394

[CR49] Nussinov R, Regev C, Jang H (2026) Energy landscapes in molecular biology: history, principles, and perspectives. Q Rev Biophys 59:e7. 10.1017/S0033583526100134 42015616

[CR50] Olerinyova A, Sonn-Segev A, Gault J, Eichmann C, Schimpf J, Kopf AH, Rudden LSP, Ashkinadze D, Bomba R, Frey L et al (2021) Mass Photometry of Membrane Proteins. Chem 7:224–236. 10.1016/j.chempr.2020.11.011 PMC7815066 33511302

[CR51] Ostrem JM, Peters U, Sos ML, Wells JA, Shokat KM (2013) K-Ras(G12C) inhibitors allosterically control GTP affinity and effector interactions. Nature 503:548–551. 10.1038/nature12796 PMC4274051 24256730

[CR52] Otzen DE, Buell AK, Jensen H (2021) Microfluidics and the quantification of biomolecular interactions. Curr Opin Struct Biol 70:8–15. 10.1016/j.sbi.2021.02.006 33831785

[CR53] Owicki JC (2000) Fluorescence polarization and anisotropy in high throughput screening: perspectives and primer. J Biomol Screen 5:297–306. 10.1177/108705710000500501 11080688

[CR54] Pascarella TW, Joseph JS, Newman JM, Borges JC, Dores-Silva PR (2026) Precision by design: surface plasmon resonance and the integration of biophysics in fragment-based drug discovery. Biophys Rev. 10.1007/s12551-026-01429-8 PMC13272721 42317548

[CR55] Pedersen ME, Gad SI, Ostergaard J, Jensen H (2019) Protein Characterization in 3D: Size, Folding, and Functional Assessment in a Unified Approach. Anal Chem 91:4975–4979. 10.1021/acs.analchem.9b00537 30916933

[CR56] Pedro L, Quinn RJ (2016) Native mass spectrometry in fragment-based drug discovery. Molecules. 10.3390/molecules21080984 PMC6274484 27483215

[CR57] Privalov PL, Potekhin SA (1986) Scanning microcalorimetry in studying temperature-induced changes in proteins. Methods Enzymol 131:4–51. 10.1016/0076-6879(86)31033-4 3773768

[CR58] Ramotowska S, Spisz P, Brzeski J, Ciesielska A, Makowski M (2022) Application of the SwitchSense technique for the study of small molecules’ (Ethidium Bromide and selected sulfonamide derivatives) affinity to DNA in real time. J Phys Chem B 126:7238–7251. 10.1021/acs.jpcb.2c03138 PMC9527753 36106569

[CR59] Renaud JP, Chung CW, Danielson UH, Egner U, Hennig M, Hubbard RE, Nar H (2016) Biophysics in drug discovery: impact, challenges and opportunities. Nat Rev Drug Discov 15:679–698. 10.1038/nrd.2016.123 27516170

[CR60] Rich RL, Myszka DG (2000) Advances in surface plasmon resonance biosensor analysis. Curr Opin Biotechnol 11:54–61. 10.1016/s0958-1669(99)00054-3 10679342

[CR61] Roberts CJ (2014) Therapeutic protein aggregation: mechanisms, design, and control. Trends Biotechnol 32:372–380. 10.1016/j.tibtech.2014.05.005 PMC4146573 24908382

[CR62] Robson-Tull J (2018) Biophysical screening in fragment-based drug design: a brief overview. Biosci Horiz 11:hzy015. 10.1093/biohorizons/hzy015

[CR63] Rout MP, Sali A (2019) Principles for integrative structural biology studies. Cell 177:1384–1403. 10.1016/j.cell.2019.05.016 PMC6810593 31150619

[CR64] Roy MJ, Winkler S, Hughes SJ, Whitworth C, Galant M, Farnaby W, Rumpel K, Ciulli A (2019) SPR-measured dissociation kinetics of PROTAC ternary complexes influence target degradation rate. ACS Chem Biol 14:361–368. 10.1021/acschembio.9b00092 PMC6423499 30721025

[CR65] Schuetz DA, de Witte WEA, Wong YC, Knasmueller B, Richter L, Kokh DB, Sadiq SK, Bosma R, Nederpelt I, Heitman LH et al (2017) Kinetics for drug discovery: an industry-driven effort to target drug residence time. Drug Discov Today 22:896–911. 10.1016/j.drudis.2017.02.002 28412474

[CR66] Seidel SA, Dijkman PM, Lea WA, van den Bogaart G, Jerabek-Willemsen M, Lazic A, Joseph JS, Srinivasan P, Baaske P, Simeonov A et al (2013) Microscale thermophoresis quantifies biomolecular interactions under previously challenging conditions. Methods 59:301–315. 10.1016/j.ymeth.2012.12.005 PMC3644557 23270813

[CR67] Soltermann F, Foley EDB, Pagnoni V, Galpin M, Benesch JLP, Kukura P, Struwe WB (2020) Quantifying protein-protein interactions by molecular counting with mass photometry. Angew Chem Int Ed Engl 59:10774–10779. 10.1002/anie.202001578 PMC7318626 32167227

[CR68] Some D, Amartely H, Tsadok A, Lebendiker M (2019) Characterization of proteins by size-exclusion chromatography coupled to multi-angle light scattering (SEC-MALS). J vis Exp. 10.3791/59615 31282880

[CR69] Stetefeld J, McKenna SA, Patel TR (2016) Dynamic light scattering: a practical guide and applications in biomedical sciences. Biophys Rev 8:409–427. 10.1007/s12551-016-0218-6 PMC5425802 28510011

[CR70] Vallat B, Webb B, Fayazi M, Voinea S, Tangmunarunkit H, Ganesan SJ, Lawson CL, Westbrook JD, Kesselman C, Sali A et al (2021) New system for archiving integrative structures. Acta Crystallogr D Struct Biol 77:1486–1496. 10.1107/S2059798321010871 PMC8647179 34866606

[CR71] Vallat B, Webb BM, Westbrook JD, Goddard TD, Hanke CA, Graziadei A, Peisach E, Zalevsky A, Sagendorf J, Tangmunarunkit H et al (2024) IHMCIF: an extension of the PDBx/mmCIF data standard for integrative structure determination methods. J Mol Biol 436:168546. 10.1016/j.jmb.2024.168546 PMC11377171 38508301

[CR72] Verkhivker GM, Agajanian S, Hu G, Tao P (2020) Allosteric regulation at the crossroads of new technologies: multiscale modeling, networks, and machine learning. Front Mol Biosci 7:136. 10.3389/fmolb.2020.00136 PMC7363947 32733918

[CR73] Wales TE, Engen JR (2006) Hydrogen exchange mass spectrometry for the analysis of protein dynamics. Mass Spectrom Rev 25:158–170. 10.1002/mas.20064 16208684

[CR74] Weikl TR, Paul F (2014) Conformational selection in protein binding and function. Protein Sci 23:1508–1518. 10.1002/pro.2539 PMC4241102 25155241

[CR75] Wen J, Lord H, Knutson N, Wikstrom M (2020) Nano differential scanning fluorimetry for comparability studies of therapeutic proteins. Anal Biochem 593:113581. 10.1016/j.ab.2020.113581 31935356

[CR76] Wilkinson MD, Dumontier M, Aalbersberg IJ, Appleton G, Axton M, Baak A, Blomberg N, Boiten JW, da Silva Santos LB, Bourne PE et al (2016) The FAIR guiding principles for scientific data management and stewardship. Sci Data 3:160018. 10.1038/sdata.2016.18 PMC4792175 26978244

[CR77] Wiseman T, Williston S, Brandts JF, Lin LN (1989) Rapid measurement of binding constants and heats of binding using a new titration calorimeter. Anal Biochem 179:131–137. 10.1016/0003-2697(89)90213-3 2757186

[CR78] Yang D, Singh A, Wu H, Kroe-Barrett R (2016) Comparison of biosensor platforms in the evaluation of high affinity antibody-antigen binding kinetics. Anal Biochem 508:78–96. 10.1016/j.ab.2016.06.024 27365220

