# Supplementary material for: The rise of state-centric biophysics: integrating orthogonal molecular readouts across biophysical scales
Source: Biophys Rev. 2026 Aug 12;18(5):1047–65. doi: 10.1007/s12551-026-01452-9 (PMC13751992; doi:10.1007/s12551-026-01452-9)
Supplement: Supplementary file 1 [file 12551_2026_1452_MOESM1_ESM.pdf]

Supplementary material

The Rise of State-Centric Biophysics: Integrating Orthogonal Molecular Readouts Across Biophysical Scales

Paulo Roberto Dores-Silva[1,2,§]; Todd Willian Pascarella[3]; Jason Michael Newman[3]; Júlio César Borges[1,§]

[1] São Carlos Institute of Chemistry, University of São Paulo, São Carlos, SP, 13566-590, Brazil; [2] Ferring Ventures, 4245 Sorrento Valley Blvd, California, United States, 92121; [3] Valenthos Biophysics, 1740 Robinson Ave, California, United States, 92103

[§] **Corresponding authors:**

Paulo Roberto Dores-Silva; Ferring Ventures Inc, 4245 Sorrento Valley Blvd – San Diego, CA 92121, USA, Phone: +1-619-770-9614; doressilvapr@gmail.com, https://orcid.org/0000-0001-8656-7769;

Júlio C. Borges; Instituto de Química de São Carlos, Universidade de São Paulo – USP, P.O. Box 780 – Zip Code 13560-970 – São Carlos, SP, Brazil, Phone: 55-16-3373-8637; Fax 55-16-3373-9982; borgesjc@iqsc.usp.br, http://orcid.org/0000-0003-4856-748X.

Abbreviation List

Abbreviation	Definition
AI	Artificial Intelligence
AUC	Analytical Ultracentrifugation
BET	Bromodomain and Extra-Terminal
BLI	Biolayer Interferometry
DLS	Dynamic Light Scattering
DSC	Differential Scanning Calorimetry
DSF	Differential Scanning Fluorimetry
E3	E3 Ubiquitin Protein Ligase
FAIR	Findable, Accessible, Interoperable, and Reusable
FIDA	Flow-Induced Dispersion Analysis
FRET	Fluorescence Resonance Energy Transfer
GDP	Guanosine Diphosphate
HDX	Hydrogen-Deuterium Exchange
HDX-MS	Hydrogen-Deuterium Exchange Mass Spectrometry
heliX	heliX® Biosensor Platform
HTS	High-Throughput Screening
IHM-CIF	Integrative/Hybrid Methods Crystallographic Information Framework
IDP	Intrinsically Disordered Protein
IM-MS	Ion Mobility Mass Spectrometry
ITC	Isothermal Titration Calorimetry
KRAS	Kirsten Rat Sarcoma Viral Oncogene Homolog
KRAS G12C	Kirsten Rat Sarcoma Viral Oncogene Homolog Gly12Cys mutant
MALS	Multi-Angle Light Scattering
MD	Molecular Dynamics
MST	Microscale Thermophoresis
MS	Mass Spectrometry
nanoDSF	Nano Differential Scanning Fluorimetry
NMR	Nuclear Magnetic Resonance
PDB	Protein Data Bank
PDB-Dev	Protein Data Bank for Integrative Structures
PROTAC	Proteolysis-Targeting Chimera
Rh	Hydrodynamic Radius
RNA	Ribonucleic Acid
SAXS	Small-Angle X-ray Scattering
SEC	Size-Exclusion Chromatography
SEC-MALS	Size-Exclusion Chromatography Coupled to Multi-Angle Light Scattering
SLS	Static Light Scattering
SPR	Surface Plasmon Resonance
TRIC	Temperature-Related Intensity Change
TDA	Taylor Dispersion Analysis
switchSENSE	switchSENSE® Dynamic Biosensor Platform
uHTS	Ultra-High-Throughput Screening

Table S1. From Isolated Measurements to Multidimensional Molecular Characterization

Period	Dominant paradigm	Representative technologies/workflows	Major advance	Primary source of confidence	Remaining limitation/transition pressure	References
1980s-mid 1990s	Single-parameter molecular characterization	ITC; DSC/scanning microcalorimetry; SPR/BIA; early optical biosensors; hydrogen-exchange/dynamics methods; DLS/AUC as solution-state sizing/assembly tools	Direct measurement of specific molecular properties such as binding constants and heats, thermal stability, real-time biospecific interactions, and protein dynamics.	Confidence primarily derived from the quality and reproducibility of an individual measurement.	Different molecular dimensions were usually measured in separate experiments, often on separate instruments.	(Batista et al., 2015; Englander and Kallenbach, 1983; Fagerstam et al., 1992; Privalov and Potekhin, 1986; Wiseman et al., 1989)
mid 1990s-2005	Throughput expansion and biosensor/fluorescence assay industrialization	Automated SPR platforms; optical biosensors; fluorescence polarization/anisotropy; FRET; robotic liquid handling; microplate readers; high-throughput screening infrastructure	Higher throughput, improved reproducibility, broader screening capacity, and more routine kinetic/affinity analysis in drug discovery environments.	Confidence derived from scale, replication, improved assay formats, and improved biosensor data quality.	Throughput increased substantially, but molecular information still remained distributed across separate assay formats.	(Leatherbarrow and Edwards, 1999; Myszka, 1999; Owicki, 2000; Rich and Myszka, 2000)
2005-2015	Sequential orthogonal characterization workflows	SPR + ITC + DSC/DSF + DLS/SEC-MALS + HDX-MS + NMR/crystallography/SAXS as complementary workflows	Routine use of independent biophysical and structural measurements to validate binding, dynamics, assembly, stability, and mechanism.	Confidence derived from agreement across independent techniques.	High protein consumption, multiple instruments, sequential workflows, and complex data reconciliation remained common.	(Konermann et al., 2011; Rich and Myszka, 2000; Some, 2013; Wales and Engen, 2006)
2010-2020	Conformation-sensitive and microfluidic/low-sample biophysics	MST; TRIC-related thermophoresis approaches; nanoDSF/backreflection; early FIDA; SEC-MALS and HDX-MS maturation; advanced SPR workflows	Greater sensitivity to solution environment, hydration shell, size, charge, conformation, thermal unfolding, aggregation, and low-sample interaction analysis.	Confidence increasingly derived from complementary readouts reporting binding-associated molecular responses.	Many molecular dimensions still required separate orthogonal experiments, even when sample consumption decreased.	(Jensen and Ostergaard, 2010; Jerabek-Willemsen et al., 2011; Kim et al., 2021; Pedersen et al., 2019)
2015-2026	Integrated multidimensional characterization platforms	Prometheus Panta; FIDA workflows; switchSENSE/heliX; Dianthus/TRIC/Spectral Shift; mass photometry; native MS; HDX-MS/native MS/structural integration	Multiple molecular dimensions increasingly obtained from the same sample, platform, or tightly coupled workflow: binding, kinetics, hydrodynamics, thermal/colloidal stability, conformational sensitivity, mass/stoichiometry, and assembly state.	Confidence derived from convergence of integrated and complementary molecular measurements.	The bottleneck shifts from data generation toward data integration, standardization, and interpretation across measurement dimensions.	(Hunter et al., 2025; Pedersen et al., 2019; Ramotowska et al., 2022)
Emerging future	State-centric multidimensional characterization	Integrated multimodal workflows combining binding, stability, hydrodynamics, conformational/oligomeric state, structural dynamics, and functional readouts	Increasing information density per experiment and tighter linkage between molecular interaction data and biological interpretation.	Confidence derived from integrated experimental context rather than isolated parameters.	Requires standardization, interoperability, rigorous controls, and transparent data-fusion strategies.	Present review; (Masson et al., 2019; Rout and Sali, 2019; Vallat et al., 2024; Wilkinson et al., 2016)

Table S2. From Single-Parameter Measurements to Multidimensional Molecular Characterization

Characterization strategy	Experimental architecture	Dominant molecular information obtained	Representative examples	Information-density logic	References
**Single-domain biophysical assays**	One technique, one dominant dimension	Affinity, thermodynamics, kinetics, stability, size, or dynamics	ITC, SPR, DSC, DLS, HDX-MS	Information concentrated in one dimension	(Batista et al., 2015; Fagerstam et al., 1992; Konermann et al., 2011; Privalov and Potekhin, 1986; Wiseman et al., 1989)
**Sequential orthogonal workflows**	Multiple experiments	Binding + stability + size + dynamics	SPR+ITC+DSF+DLS+HDX-MS	Higher information through integration across experiments	(Konermann et al., 2011; Pedro and Quinn, 2016; Some et al., 2019; Some and Razinkov, 2019)
**Conformation-sensitive and microfluidic methods**	Single assay reports binding and environmental changes	Binding plus conformational/hydrodynamic sensitivity	MST, TRIC, FIDA, switchSENSE	More dimensions per experiment	(Asor and Kukura, 2022; Jerabek-Willemsen et al., 2011; Otzen et al., 2021; Pedersen et al., 2019; Seidel et al., 2013)
**Integrated multidimensional platforms**	Multiple readouts in same platform	Binding, stability, aggregation, hydrodynamics, assembly state	Prometheus Panta, FIDA, heliX, Dianthus, mass photometry	High information density	(Asor and Kukura, 2022; Clery et al., 2017; Jerabek-Willemsen et al., 2011; Kim et al., 2021; Otzen et al., 2021; Pedersen et al., 2019; Ramotowska et al., 2022; Seidel et al., 2013)
**State-centric characterization workflows**	Integrated workflows	Binding interpreted with molecular state	Integrated SPR/HDX-MS/native MS/Prometheus/TRIC workflows	Highest conceptual information density	Review synthesis

Table S3. Molecular dimensions captured by representative integrated biophysical platforms.

Platform/workflow	Primary principle	Affinity/binding	Kinetics	Thermal/ colloidal stability	Hydrodynamics/size	Conformation/ structural dynamics	Oligomeric/ assembly state	Throughput/sample economy	Interpretation caution	References
**High-throughput label-free biosensors (SPR / array SPR / BLI / related optical biosensors)**	Real-time label-free monitoring of biomolecular association and dissociation at a sensor surface or biosensor tip/array.	Direct: KD/affinity can be derived from equilibrium or kinetic analysis.	Direct: kon, koff, and residence-time information are core outputs for SPR and related biosensors.	Not primary.	Not primary.	Indirect/conditional: complex sensorgrams can suggest heterogeneity or conformational effects, but structural interpretation requires orthogonal support.	Indirect/conditional: epitope binning, competition, and avidity analysis can inform assembly/interaction architecture but do not directly measure oligomeric state.	High for modern parallel/array formats; throughput varies strongly by instrument and assay design.	Surface immobilization, mass transport, rebinding, avidity, matrix effects, and model selection can bias interpretation.	(Knowling et al., 2020; Rich and Myszka, 2000; Yang et al., 2016)
**FIDA / TDA-based flow-induced dispersion workflows**	Capillary flow-induced dispersion/Taylor dispersion analysis measures apparent hydrodynamic radius or diffusivity changes under native solution conditions.	Direct/derived: noncovalent interactions and affinity can be quantified from binding-dependent size changes.	Not primary in the core cited FIDA papers used here.	Conditional: protein folding/stability assessment is supported in integrated TDA/FIDA/in-line fluorescence workflows.	Direct: hydrodynamic radius / apparent size is the central readout.	Conditional: conformational/ folding changes can be inferred when coupled to size and intrinsic fluorescence measurements.	Conditional: oligomerization or complex formation can be inferred from size/species changes when assay design supports it.	Low-volume/nanoliter to microliter solution measurements; generally sample-sparing relative to many conventional workflows.	Requires a detectable size/diffusivity change and appropriate assay design; labeling/indicator strategy may influence interpretation.	(Jensen and Ostergaard, 2010; Otzen et al., 2021; Pedersen et al., 2019)
**switchSENSE / helix**	Electro-switchable DNA nanolevers with fluorescence readout; static and dynamic modes report binding and nanolever motion.	Direct: KD/affinity can be determined for suitable immobilized systems.	Direct: association and dissociation kinetics are reported.	Conditional: thermal stability or melting behavior can be measured in specific switchSENSE assay formats, but not the universal core output.	Direct: nanolever dynamics report hydrodynamic drag and can yield hydrodynamic diameter/size.	Direct/conditional: conformational changes can be detected through altered switching dynamics or fluorescence proximity response.	Conditional: ternary/multivalent complexes can be characterized in appropriate assay formats.	Moderate/high depending on chip format and assay design; integrated multiparameter characterization in one platform.	Requires DNA-nanolever attachment/immobilization strategy; surface geometry and tethering must be considered.	(BrukerDaltonics, 2025; Clery et al., 2017; Langer et al., 2013)
**Prometheus Panta / nanoDSF + backreflection + DLS/SLS**	Intrinsic fluorescence nanoDSF combined with light-scattering/backreflection modalities for stability and	Indirect only: ligand binding may be inferred from stabilization/thermal shift, but direct affinity determination requires dose-	Not primary.	Direct: nanoDSF reports conformational thermal stability; backreflection/light scattering reports	Direct: DLS/SLS provide particle-size/light-scattering information when equipped/configured.	Indirect/conditional: unfolding profiles and fluorescence ratios report folding-state changes, not residue-level	Conditional: DLS/SLS/backreflection can detect aggregation or particle populations, but not high-	Moderate/high: capillary and multi-sample formats; integrated stability/sizing readouts reduce separate assays.	Thermal shifts are not automatically equivalent to binding affinity; aggregation and irreversibility can complicate interpretation.	(Kim et al., 2021; NanoTemperTechnologies, 2025b; Stetefeld et al., 2016)

	particle-size characterization.	response assumptions or orthogonal methods.		aggregation/colloidal behavior; DLS/SLS support particle sizing/scattering.		structural dynamics.	resolution stoichiometry.			
**Dianthus/ TRIC/ Spectral Shift**	Fluorescence-based affinity screening using Temperature-Related Intensity Change and/or Spectral Shift in plate-based formats.	Direct/derived: KD/affinity can be determined from concentration-dependent fluorescence changes.	Not primary in standard endpoint affinity-screening use.	Not primary.	Not primary.	Indirect/conditional: fluorescence changes report local dye environment and may be sensitive to binding-associated molecular changes.	Conditional: can study protein-ligand, protein-protein, protein-nucleic acid, and ternary-complex interactions, but does not directly measure oligomeric mass/state.	High: plate-based and uHTS implementations support large-scale screening with low assay volumes.	Requires labeling or dye strategy; fluorescence artifacts, aggregates, and dye-environment effects require controls.	(Hunter et al., 2025; Langer et al., 2022; NanoTemperTechnologies, 2025a)
**Mass photometry**	Label-free optical single-molecule mass measurement in solution via interferometric scattering.	Direct/derived: affinities can be estimated from relative abundance/speciation under equilibrium conditions.	Conditional: dynamics/on-off rates can be accessed in suitable time-resolved or abundance-based designs.	Not primary.	Not hydrodynamic size; measures molecular mass distributions rather than Rh.	Indirect/conditional: dynamics and conformational effects may influence species distributions but are not residue-level structural dynamics.	Direct: mass distributions reveal oligomeric state, complex formation, heterogeneity, and relative abundance.	Low sample consumption and rapid measurements; concentration window and surface interactions must be considered.	Requires mass contrast/resolution and suitable concentration regime; weak/fast equilibria may require specialized workflows.	(Asor and Kukura, 2022; Asor et al., 2025; Olerinyova et al., 2021) (Kofinova et al., 2024)
**Native mass spectrometry / ion mobility-native MS**	Mass spectrometry under native-like conditions to preserve noncovalent complexes; ion mobility can add gas-phase shape/collision cross-section information.	Direct/derived: protein-ligand binding and affinity ranking/quantification can be obtained for suitable systems.	Conditional: kinetic information is possible in specialized workflows but not the primary generic output.	Not primary for thermal/colloidal stability.	Not solution hydrodynamic size; IM-MS can provide collision cross section and conformer/shape information in gas phase.	Direct/conditional: ion mobility and native MS can report conformational/ structural heterogeneity of complexes.	Direct: stoichiometry, complex composition, ligand occupancy, and assembly state are core outputs.	Moderate/high for screening workflows depending on automation and sample prep.	Gas-phase transfer, ionization, adducts, and preservation of native-like states must be carefully controlled.	(Gavriilidou et al., 2022; Pedro and Quinn, 2016; Tamara et al., 2022)
**HDX-MS**	Hydrogen/deuterium exchange coupled to MS reports backbone amide exchange as a function of solvent accessibility and dynamics.	Indirect: can map binding effects and interaction regions, but does not directly yield KD without separate titration/modeling.	Indirect/conditional: exchange kinetics report protein dynamics, not binding association/dissociation kinetics.	Conditional: can report stabilization/protection patterns, but not a primary thermal-stability assay.	Not hydrodynamic size.	Direct: structural dynamics, conformational protection/deprotection, solvent accessibility, and allosteric changes.	Conditional: can support assembly/interface mapping but does not directly measure oligomeric mass.	Moderate throughput; increasingly automated workflows but generally lower throughput than plate-based screening.	Spatial resolution, back-exchange, peptide coverage, uptake interpretation, and ensemble averaging limit atomistic conclusions.	(Englander and Kallenbach, 1983; Konermann et al., 2011; Wales and Engen, 2006)

References

Asor, R, Kukura, P (2022). Characterising biomolecular interactions and dynamics with mass photometry. Curr Opin Chem Biol 68:102132. 10.1016/j.cbpa.2022.102132.

Asor, R, Loewenthal, D, van Wee, R, Benesch, JLP, Kukura, P (2025). Mass Photometry. Annu Rev Biophys 54:379-399. 10.1146/annurev-biophys-061824-111652.

Batista, FA, Gava, LM, Pinheiro, GM, Ramos, CH, Borges, JC (2015). From Conformation to Interaction: Techniques to Explore the Hsp70/Hsp90 Network. Current protein & peptide science 16:735-753. 10.2174/1389203716666150505225744.

BrukerDaltonics (2025). switchSENSE®: Dynamic interaction analysis and molecular characterization. https://www.bruker.com/en/products-and-solutions/biosensors-for-molecular-interactions/switchsense-technology.html. Accessed 06252026.

Clery, A, Sohier, TJM, Welte, T, Langer, A, Allain, FHT (2017). switchSENSE: A new technology to study protein-RNA interactions. Methods 118-119:137-145. 10.1016/j.ymeth.2017.03.004.

Englander, SW, Kallenbach, NR (1983). Hydrogen exchange and structural dynamics of proteins and nucleic acids. Q Rev Biophys 16:521-655. 10.1017/s0033583500005217.

Fagerstam, LG, Frostell-Karlsson, A, Karlsson, R, Persson, B, Ronnberg, I (1992). Biospecific interaction analysis using surface plasmon resonance detection applied to kinetic, binding site and concentration analysis. J Chromatogr 597:397-410. 10.1016/0021-9673(92)80137-j.

Gavriilidou, AFM, Sokratous, K, Yen, HY, De Colibus, L (2022). High-Throughput Native Mass Spectrometry Screening in Drug Discovery. Front Mol Biosci 9:837901. 10.3389/fmolb.2022.837901.

Hunter, CE, Pohl, E, Freitag-Pohl, S (2025). High-throughput investigation of macromolecular interactions for drug development using spectral shift technology. Biophys Rev 17:959-980. 10.1007/s12551-025-01359-x.

Jensen, H, Ostergaard, J (2010). Flow induced dispersion analysis quantifies noncovalent interactions in nanoliter samples. J Am Chem Soc 132:4070-4071. 10.1021/ja100484d.

Jerabek-Willemsen, M, Wienken, CJ, Braun, D, Baaske, P, Duhr, S (2011). Molecular interaction studies using microscale thermophoresis. Assay Drug Dev Technol 9:342-353. 10.1089/adt.2011.0380.

Kim, SH, Yoo, HJ, Park, EJ, Na, DH (2021). Nano Differential Scanning Fluorimetry-Based Thermal Stability Screening and Optimal Buffer Selection for Immunoglobulin G. Pharmaceuticals (Basel) 15. 10.3390/ph15010029.

Knowling, S, Clark, J, Sjuts, H, Abdiche, YN (2020). Direct Comparison of Label-Free Biosensor Binding Kinetics Obtained on the Biacore 8K and the Carterra LSA. SLAS Discov 25:977-984. 10.1177/2472555220934814.

Kofinova, Z, Karunanithy, G, Ferreira, AS, Struwe, WB (2024). Measuring Protein-Protein Interactions and Quantifying Their Dissociation Constants with Mass Photometry. Curr Protoc 4:e962. 10.1002/cpz1.962.

Konermann, L, Pan, J, Liu, YH (2011). Hydrogen exchange mass spectrometry for studying protein structure and dynamics. Chem Soc Rev 40:1224-1234. 10.1039/c0cs00113a.

Langer, A, Bartoschik, T, Cehlar, O, Duhr, S, Baaske, P, Streicher, W (2022). A New Spectral Shift-Based Method to Characterize Molecular Interactions. Assay Drug Dev Technol 20:83-94. 10.1089/adt.2021.133.

Langer, A, Hampel, PA, Kaiser, W, Knezevic, J, Welte, T, Villa, V, Maruyama, M, Svejda, M, Jahner, S, Fischer, F et al. (2013). Protein analysis by time-resolved measurements with an electro-switchable DNA chip. Nat Commun 4:2099. 10.1038/ncomms3099.

Leatherbarrow, RJ, Edwards, PR (1999). Analysis of molecular recognition using optical biosensors. Curr Opin Chem Biol 3:544-547. 10.1016/s1367-5931(99)00006-x.
Masson, GR, Burke, JE, Ahn, NG, Anand, GS, Borchers, C, Brier, S, Bou-Assaf, GM, Engen, JR, Englander, SW, Faber, J et al. (2019). Recommendations for performing, interpreting and reporting hydrogen deuterium exchange mass spectrometry (HDX-MS) experiments. Nat Methods 16:595-602. 10.1038/s41592-019-0459-y.
Myszka, DG (1999). Improving biosensor analysis. J Mol Recognit 12:279-284. 10.1002/(SICI)1099-1352(199909/10)12:5<279::AID-JMR473>3.0.CO;2-3.
NanoTemperTechnologies (2025a). Dianthus—TRIC® and Spectral Shift Technology. https://nanotempertech.com/dianthus/. Accessed 06252026.
NanoTemperTechnologies (2025b). Prometheus™ Panta: One readout. Multiple insights. https://nanotempertech.com/prometheus/?utm. Accessed 06252026.
Olerinyova, A, Sonn-Segev, A, Gault, J, Eichmann, C, Schimpf, J, Kopf, AH, Rudden, LSP, Ashkinadze, D, Bomba, R, Frey, L et al. (2021). Mass Photometry of Membrane Proteins. Chem 7:224-236. 10.1016/j.chempr.2020.11.011.
Otzen, DE, Buell, AK, Jensen, H (2021). Microfluidics and the quantification of biomolecular interactions. Curr Opin Struct Biol 70:8-15. 10.1016/j.sbi.2021.02.006.
Owicki, JC (2000). Fluorescence polarization and anisotropy in high throughput screening: perspectives and primer. J Biomol Screen 5:297-306. 10.1177/108705710000500501.
Pedersen, ME, Gad, SI, Ostergaard, J, Jensen, H (2019). Protein Characterization in 3D: Size, Folding, and Functional Assessment in a Unified Approach. Anal Chem 91:4975-4979. 10.1021/acs.analchem.9b00537.
Pedro, L, Quinn, RJ (2016). Native Mass Spectrometry in Fragment-Based Drug Discovery. In Molecules, p. 984.
Privalov, PL, Potekhin, SA (1986). Scanning microcalorimetry in studying temperature-induced changes in proteins. In Methods in Enzymology (Academic Press), pp. 4-51.
Ramotowska, S, Spisz, P, Brzeski, J, Ciesielska, A, Makowski, M (2022). Application of the SwitchSense Technique for the Study of Small Molecules' (Ethidium Bromide and Selected Sulfonamide Derivatives) Affinity to DNA in Real Time. J Phys Chem B 126:7238-7251. 10.1021/acs.jpcb.2c03138.
Rich, RL, Myszka, DG (2000). Advances in surface plasmon resonance biosensor analysis. Curr Opin Biotechnol 11:54-61. 10.1016/s0958-1669(99)00054-3.
Rout, MP, Sali, A (2019). Principles for Integrative Structural Biology Studies. Cell 177:1384-1403. 10.1016/j.cell.2019.05.016.
Seidel, SA, Dijkman, PM, Lea, WA, van den Bogaart, G, Jerabek-Willemsen, M, Lazic, A, Joseph, JS, Srinivasan, P, Baaske, P, Simeonov, A et al. (2013). Microscale thermophoresis quantifies biomolecular interactions under previously challenging conditions. Methods 59:301-315. 10.1016/j.ymeth.2012.12.005.
Some, D (2013). Light-scattering-based analysis of biomolecular interactions. Biophys Rev 5:147-158. 10.1007/s12551-013-0107-1.
Some, D, Amartely, H, Tsadok, A, Lebendiker, M (2019). Characterization of Proteins by Size-Exclusion Chromatography Coupled to Multi-Angle Light Scattering (SEC-MALS). J Vis Exp. 10.3791/59615.
Some, D, Razinkov, V (2019). High-Throughput Analytical Light Scattering for Protein Quality Control and Characterization. Methods Mol Biol 2025:335-359. 10.1007/978-1-4939-9624-7_16.

Stetefeld, J, McKenna, SA, Patel, TR (2016). Dynamic light scattering: a practical guide and applications in biomedical sciences. Biophys Rev 8:409-427. 10.1007/s12551-016-0218-6.

Tamara, S, den Boer, MA, Heck, AJR (2022). High-Resolution Native Mass Spectrometry. Chem Rev 122:7269-7326. 10.1021/acs.chemrev.1c00212.

Vallat, B, Webb, BM, Westbrook, JD, Goddard, TD, Hanke, CA, Graziadei, A, Peisach, E, Zalevsky, A, Sagendorf, J, Tangmunarunkit, H et al. (2024). IHMCIF: An Extension of the PDBx/mmCIF Data Standard for Integrative Structure Determination Methods. J Mol Biol 436:168546. 10.1016/j.jmb.2024.168546.

Wales, TE, Engen, JR (2006). Hydrogen exchange mass spectrometry for the analysis of protein dynamics. Mass Spectrom Rev 25:158-170. 10.1002/mas.20064.

Wilkinson, MD, Dumontier, M, Aalbersberg, IJ, Appleton, G, Axton, M, Baak, A, Blomberg, N, Boiten, JW, da Silva Santos, LB, Bourne, PE et al. (2016). The FAIR Guiding Principles for scientific data management and stewardship. Sci Data 3:160018. 10.1038/sdata.2016.18.

Wiseman, T, Williston, S, Brandts, JF, Lin, LN (1989). Rapid measurement of binding constants and heats of binding using a new titration calorimeter. Anal Biochem 179:131-137. 10.1016/0003-2697(89)90213-3.

Yang, D, Singh, A, Wu, H, Kroe-Barrett, R (2016). Comparison of biosensor platforms in the evaluation of high affinity antibody-antigen binding kinetics. Anal Biochem 508:78-96. 10.1016/j.ab.2016.06.024.
